# Electromagnetic imaging reveals insufficient fluids to explain shallow megathrust creep at the Shumagin Gap

**DOI:** 10.1038/s41467-026-71176-7

**Published:** 2026-03-28

**Authors:** Yinchu Li, Samer Naif, Kerry Key, Steven Constable, Rob L. Evans, Donna J. Shillington, Anne Bécel, Darcy Cordell

**Affiliations:** 1 School of Earth and Atmospheric Sciences, Georgia Institute of Technology, Atlanta, GA USA; 2 Deep Blue Geophysics, LLC, Los Angeles, CA USA; 3 Scripps Institution of Oceanography, La Jolla, CA USA; 4 Department of Geology and Geophysics, Woods Hole Oceanographic Institution, Woods Hole, MA USA; 5 School of Earth and Sustainability, Northern Arizona University, Flagstaff, AZ USA; 6 Lamont-Doherty Earth Observatory, Columbia University, Palisades, NY USA; 7 Centre for Science, Athabasca University, Athabasca, Alberta Canada; 8 Department of Physics, University of Alberta, Edmonton, Alberta Canada

**Keywords:** Geophysics, Tectonics, Geodynamics

## Abstract

The Shumagin Gap, a creeping segment of the Alaska subduction zone characterized by tsunamigenic structures, experienced a deep rupture during the July 2020 M7.8 earthquake. However, shallow slip behavior and the upper boundary of the rupture remain poorly understood. Here we utilize controlled-source electromagnetic data to image subsurface electrical resistivity, investigating the role of fluids in modulating megathrust locking state within the Shumagin Gap. Results reveal pronounced trench-normal heterogeneity in electrical resistivity both along the shallow plate interface and within the overriding plate, showing fluid presence but low overall porosity at the interface. An observed conductive channel extending into the overriding plate may facilitate upward fluid drainage. Our findings suggest that the volumes of fluids and inferred pore pressures are not sufficient to explain megathrust creep at the Shumagin Gap. Rather, the intricate interplay between heterogeneous structure and fluid distribution contributes to the region’s seismogenic behavior and tsunami hazards, particularly in the shallow portion of the megathrust.

## Abstract (web summary)

Controlled-source Electromagnetic imaging of the Shumagin Gap shows strong resistivity heterogeneity, with limited fluid volume and low inferred pore pressure along the shallow megathrust, suggesting that structural complexity, not fluid overpressure alone, governs creeping behavior.

## Introduction

Understanding the key mechanisms that govern megathrust slip behavior and seismic activity is essential for advancing our knowledge of subduction zone processes and assessing related seismic hazards. Fluids have been thought to profoundly influence or control many subduction zone processes^1–5^. Fluids are primarily released from sediment compaction in the outermost forearc^6^ and through dehydration reactions farther downdip^7^. Along megathrust faults, these fluids affect the mechanical behavior of faults by altering pore pressure^1^. One prevailing hypothesis suggests that excess pore pressure along the megathrust reduces the effective normal stress and therefore promotes creep^8,9^. Yet, the extent to which fluid-driven processes influence plate interface slip behavior remains poorly understood.

**Fig. 1:**
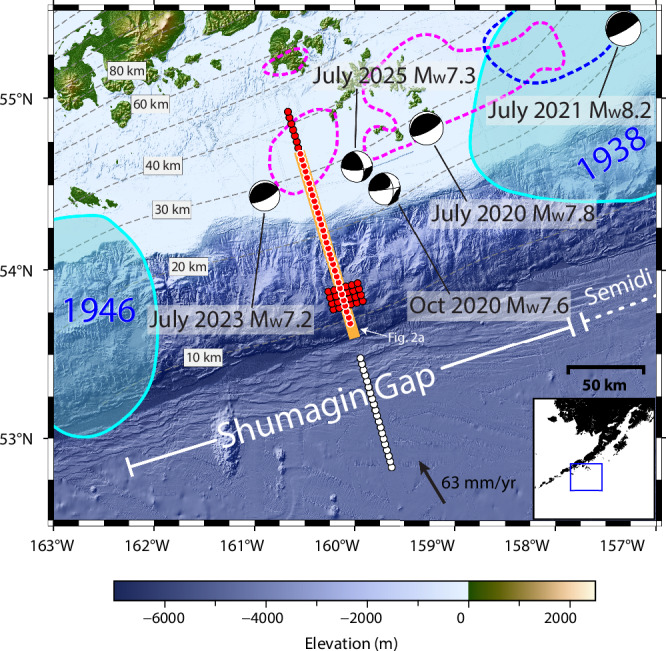
Tectonic setting of the study region and the ElectroMagnetic Alaskan GeoPRISMS Experiment (EMAGE) survey profile in the Shumagin Gap. The magenta dashed line outlines the July 2020 Mw 7.8 earthquake rupture area with coseismic slip $\ge$ 0.5 m^19^, and the blue dashed line denotes the July 2021 Mw 8.2 earthquake rupture area with coseismic slip $\ge$ 2 m^70^. Focal mechanisms are from GCMT^71^. Red circles mark ocean-bottom electromagnetic (OBEM) receivers recording both MT and CSEM signals deployed during the EMAGE experiment; white circles seaward of the trench mark the MT-only receivers. An additional array of CSEM data was collected on the forearc slope of the Shumagin Gap for future 3D imaging. Red circles with white outlines indicate the CSEM receivers used in this study. The thick orange line marks the location and extent of the cross-section shown in Fig. 2a. Slab depths below sea level from the Slab2.0 model^72^ are shown with gray dashed lines at 10 km intervals. The average convergence rate between the Pacific plate and the North American plate is ~63 mm/yr^73^. Cyan-shaded patches highlight historical great megathrust earthquake ruptures^12^. The inset shows the map location of the study region. Base map generated with Generic Mapping Tools^74^ from the Global Multi-Resolution Topography (GMRT) synthesis^75^ v4.4 grid data.

The Shumagin Gap of the Alaska-Aleutian Subduction Zone (AASZ) offers an ideal target to investigate the relationship between fluids and the nature of megathrust slip (Fig. 1). This region behaves differently from its neighboring segments^2,10,11^ and is recognized as a seismic gap because no significant earthquakes (Mw > 8) have occurred for at least a century^12^. Specifically, the Shumagin Gap is distinguished by abundant interplate and intermediate-depth seismicity^2^, extensive faulting associated with plate bending^2,13^, a rugged lower-plate relief, and a sediment-starved trench^2,3,14^. Despite the lack of prominent seamounts on the incoming plate and the absence of clearly resolved subducted seamounts, the plate interface remains highly rugged, characterized by extensive topographic relief associated with bend faulting. In addition, geodetic observations show a weakly locked plate interface in this region^10^. Past studies present conflicting evidence regarding fluid accumulation in the Shumagin Gap. Seismic imaging suggests that intense bend faulting in the incoming plate promotes hydration in the crust and upper mantle and introduces a substantial amount of fluid to the system^2,15,16^. Yet this additional fluid likely impacts intraslab seismicity and subduction processes farther downdip, rather than influencing effective stress and fault creep within the seismogenic zone^2,17^. Multi-channel seismic reflection data have revealed thin and irregular subducted sediments, leading to the inference of low pore fluid pressure in the shallow portion (within 12 km of the trench) of the Shumagin Gap^3^. Furthermore, magnetotelluric (MT) data used to image the deeper resistivity structure beneath the forearc shelf^18^ observed a similarly fluid-starved plate interface at depths shallower than 25 km (between 60 and 90 km from the trench), with a segment of larger fluid accumulations at greater depths (100–140 km from the trench). In contrast, Vp/Vs ratios at 15–35 km depth have been used to infer that both the plate interface and overriding plate are fluid-rich throughout the Shumagin Gap^5^, although the shallow portion (<10 km) of the megathrust near the trench remains poorly imaged. Contributing further complexity is the rugged plate interface geometry seen in seismic reflection imaging^3,14^, which might be causally related to fault creep. In addition, the slip behavior and degree of geodetic locking of the shallow plate interface remains enigmatic^5,19–22^, with incomplete understanding of along dip variations in slip behavior.

The recent sequence of seismic events highlights the complex along-strike segmentation in megathrust locking and slip behavior within the AASZ. The July 2020 M7.8 Simeonof earthquake initiated near the border between the Semidi segment and Shumagin Gap and propagated westward to the eastern Shumagin Gap^23^, but ruptured the deeper portion of the seismogenic zone and therefore only partially filled the gap^11^. This event was followed by an unusual M7.6 strike-slip intraplate earthquake in October 2020 that also involved slip on the megathrust and/or an upper plate fault^24,25^. Subsequently, an M8.2 megathrust earthquake struck to the east of the Shumagin Gap on 29 July 2021, primarily rupturing the western part of the 1938 M8.2 rupture zone. This event exhibited distinct slip distributions and seismic characteristics, indicating that it was not a repeat of the 1938 earthquake^26^.

According to MT imaging, the July 2020 rupture propagated through a deeper, fluid-rich segment of the plate interface around 30 km depth, rather than rupturing updip into more resistive, fluid-poor areas^18^, possibly because the fluid-rich portion of the plate interface is conditionally stable^20,27^. These fluid-rich regions, where high pore pressures may exist, can promote stable fault creep during interseismic periods. However, they can become unstable and experience dynamic rupture when triggered by nearby stress concentrations that initiated rupture elsewhere.

The structural and compositional properties of the overriding plate also appear to affect megathrust slip behavior^28–30^. In the Shumagin Gap, an active deep-rooted normal fault separates the Cretaceous Chugach Terrane from the younger, Paleocene Prince William Terrane (PWT), potentially impacting tsunami hazards^14^. Variations in the seismic velocity of the overriding plate also suggest changes in rigidity that could influence megathrust slip behavior, segmentation, and seismicity patterns^30,31^. The overriding forearc crust offers key insights into fluid release and redistribution, fluid-driven fault weakening, and pore pressure variations^4,32,33^. Understanding the fate of these fluids within the forearc crust is especially critical for constraining shallow slip behavior and tsunamigenesis.

In this study, we use controlled-source electromagnetic (CSEM) data to image the electrical resistivity of the shallow megathrust and overriding plate in the Shumagin Gap. Our CSEM profile extends from ~10 to 120 km inboard of the trench axis, revealing the resistivity structure of the shallow megathrust updip of the collocated MT study^18^ and providing higher resolution imaging in the upper plate. Electrical resistivity is notably sensitive to fluids (e.g., Naif et al.^34^) and hence serves as a powerful proxy for fluid volume and distribution at depth. Our inverted resistivity model and subsequent porosity estimates and analyses identify a shallow, fluid-poor plate interface with low fluid pressures, indicating that high pore pressure does not fully account for its creeping behavior. Instead, slip mode and seismic hazard in this region are likely governed by the interplay among multiple factors, such as limited fluid availability along the plate interface, interface roughness, and heterogeneities in the rigidity, stress state, and permeability of the overriding plate (e.g., Bassett et al.^35^). By offering constraints on near-surface fluid distributions and bridging the gap between prior seismic and geodetic observations, the CSEM data can advance our understanding of how along-dip changes in fluids, structure, and slip behavior influence both seismic hazard and the broader subduction system.

## Results

### Heterogeneous outer forearc and shelf electrical structure

**Fig. 2:**
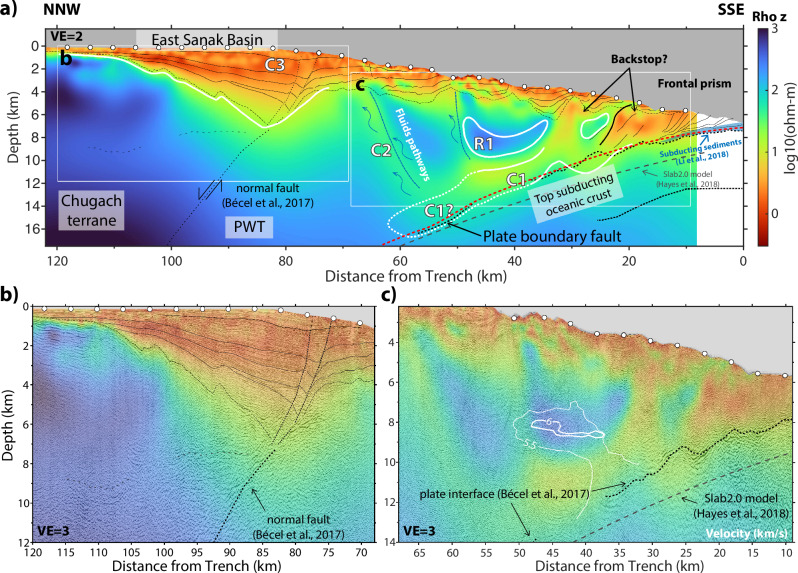
Electrical resistivity model and integrated interpretation of the Shumagin Gap outer shelf and forearc. **a** Vertical electrical resistivity model, showing key conductive and resistive features of the margin. Structural interpretations indicated by black dashed and solid lines are from seismic reflection imaging^14^, highlighting the East Sanak sedimentary basin, plate boundary fault, and a deep-rooted active normal fault separating the Prince William Terrane (PWT) from the Chugach terrane. White lines delineate geological features revealed by CSEM data, such as a conductive layer above the shallow plate interface (C1), the East Sanak Basin (C3), and relatively high-resistivity bodies (e.g., R1) in the outer wedge. Blue dashed lines and blue arrows indicate possible seaward-dipping faults behaving as fluid pathways (e.g., C2) that contribute to upper plate heterogeneity. The gray dashed line shows the interface extracted from the Slab2.0 model^72^. The red dashed line represents the plate interface approximated from seismic reflection imaging^14^, along which porosity is estimated in subsequent analyses. The region circled by the white dashed line is possibly a downdip extension of the conductive layer beyond the CSEM data sensitivity. **b,** **c** Co-located seismic reflection images from profile ALEUT Line 5^14^ overlain on the resistivity model. A localized high-velocity anomaly (Vp > 5.5 km/s) from the P-wave velocity model^30^ is contoured in white in (**c**) and correlates spatially with the resistive body R1. White circles on the seafloor represent ocean-bottom electromagnetic (OBEM) receivers. There were no receivers or towed transmitter data available within ~10 km landward of the trench. Boxes in (**a**) indicate the location of (**b**) on the shelf and the location of **c** in the forearc. Vertical exaggeration is 2× in (**a**) and 3× in (**b,** **c**). The resistivity and seismic models without interpretation are shown in Fig. S1.

We present our preferred resistivity model obtained through vertically transverse anisotropic modeling of the CSEM data collected along the survey line in this study (Figs. 1 and 2, “Methods”). In the outer forearc, a dipping conductive zone (C1) extends from the frontal prism to approximately 50 km landward of the trench at about 10 km below the seafloor. The along-dip variation in electrical resistivity suggests scattered patches of moderately conductive structure along the plate interface, likely reflecting irregularities at the plate interface and heterogeneity along the subduction zone boundary. Given the paucity of subducting sediments^3,14^ and the abundant bending faults in the incoming plate^2,13^, the observed elevated conductivity along the slab perhaps implies a complex interaction between subducting and overriding plates, in which fluids derived from the subducting oceanic crust can migrate into and accumulate within the overlying forearc crust.

Close to the trench, a small and relatively high-conductivity frontal prism is bounded by possible backstop splay fault zones. Such structures can be prone to tsunamigenesis^36^. Farther landward, between the East Sanak Basin and the frontal prism, conductive channels cut across the resistive margin framework. These channels suggest the presence of localized permeable zones, possibly related to faults, that act as fluid pathways from the plate interface up into the upper plate, similar to conductive features observed on the Nicaragua forearc slope^37^. One notable conductive channel appears at 50–70 km landward of the trench (C2), stretching from the downdip edge of C1 to the seafloor slope sediments. Given the complex near-surface electrical resistivity structures in this forearc slope, additional geochemical sampling of fault-associated seeps is needed to determine whether slab fluids escape to the seafloor through this channel. This large, seaward-dipping conductive zone (C2) also separates the electrically heterogeneous, relatively shallow framework material from a deeper, more resistive Prince William Terrane (PWT). A localized high-velocity anomaly (Vp > 5.5 km/s) delineated by white contours in Fig. 2c^30^ spatially aligns with a high-resistivity anomaly (R1). The sharp contrast between R1 and conductive frontal prism suggests a boundary separating the strong, older framework materials from the younger frontal prism. The geometry of the East Sanak Basin, which thins to the north, is well resolved in our resistivity model (C3; Fig. 2a, b). This thick (~6 km) and electrically conductive sedimentary basin attenuates the electromagnetic signal penetration, reducing the data sensitivity and resolution to deeper structure below the basin (Methods). This limits our ability to determine whether fluids are associated with the large normal fault (Fig. 2a, b), which extends upwards from the plate interface and separates Chugach and Prince William Terranes, though this fault does extend into the East Sanak Basin and offsets several sedimentary units as seen by active-source seismic reflection imaging^14^.

### Low porosity and fluid pressure along the plate interface

**Fig. 3:**
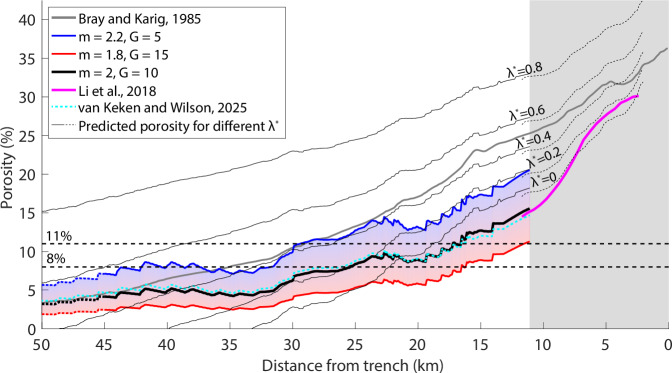
Estimated porosity along the plate interface using Archie’s law. The thick black line shows the porosity derived from Archie’s law with a cementation exponent of $m$ = 2 and a linear geothermal model with thermal gradient *G* = 10 °C km^−1^). Red and blue curves represent two end-member scenarios ($m$ = 2.2, *G* = 5 °C km^−1^ and $m$ = 1.8, *G* = 15 °C km^−1^) considering the wide range of uncertainties (blue-red shaded area) in thermal structure and sediment consolidation in this study. The magenta line shows porosity converted from collocated seismic observations^3^. For comparison, thin black lines represent predicted porosities for different fluid pressure ratios λ* (0, 0.2, 0.4, 0.6, and 0.8; “Methods”). The dashed segments of these lines are converted from collocated seismic results^3^. The gray line represents a generic compaction trend^41^ as a lower bound. The dashed cyan line shows porosity estimated along the plate interface using the temperature from van Keken and Wilson^67^. Two horizontal black dashed lines indicate the average porosity of layer 2A of the incoming oceanic crust along ALEUT Line 5^2^ before bending (8%) and after bending (11%)^15^. Dashed segments beyond 45 km landward indicate regions where CSEM sensitivity to the plate interface diminishes. We exclude estimates within 11 km of the trench (shaded area) due to a lack of data coverage in deep water.

We apply the empirical Archie’s law^38^ to estimate the porosity from our preferred electrical resistivity model (Figs. 2 and S2, “Methods”). We show the estimated along-dip porosity under three scenarios of thermal gradient ($G$) and cementation exponent ($m$) in Fig. 3. Beyond ~10 km landward of the trench, the baseline estimate ($m$ = 2, *G* = 10 °C km^−1^) shows that the maximum porosity stays around 15% at approximately 2 km below the seafloor. Even for the upper bound porosity endmember ($m$ = 2.2, *G* = 5 °C km^−1^), our estimate remains below 20%. Within ~10 km landward of the trench, we did not deploy receivers, and water depths exceeded the maximum operational rating of our deep-towed electromagnetic transmitter system. Consequently, data coverage is insufficient to resolve plate interface porosity in the near-trench region. However, seismic reflection data^3^ suggest only thin or highly compacted sediments with low porosity and low pore pressures subducting in this region. Furthermore, porosity inferred from pre-stack depth migration-derived P-wave velocity^39^ in the Shumagin Gap agrees well with our CSEM-based estimates at around 11 km landward of the trench^3^. This independent observation lends confidence to our inferred porosity trend farther downdip from the near-trench region in this study. Combining the near-trench porosity estimates from P-wave velocity^3^ with our observations beyond ~10 km, the porosity estimates in the near-trench region are considerably lower than the compaction trends of many other subduction zones at comparable depths^40^ and even from the neighboring Semidi segment^3^. Furthermore, extrapolating a global generic porosity compaction trend^41^ towards the seafloor as a lower bound still results in higher porosity values than the near-trench estimates obtained for this region.

To investigate the range of pore pressures that are consistent with our porosity estimates, we follow Li et al.^3^ and compare our estimated porosities with those predicted from sediment consolidation for a range of fluid pressure ratios λ* (0, 0.2, 0.4, 0.6, and 0.8) in Fig. 3 (“Methods”), where the value of zero represents hydrostatic fluid pressure. For the near-trench region where our data coverage is limited, we convert the predicted P-wave velocities from Li et al.^3^ into porosities using the relationship of Hoffman & Tobin^39^, for the same range of assumed pore pressure ratios. The nearly identical porosity estimates from seismic and CSEM at 12 km landward of the trench highlight how our work complements the findings of Li et al.^3^. Comparing the predicted porosities for different fluid pressures with the porosity estimated from our resistivity model demonstrates that the shallow plate interface in the Shumagin Gap is primarily consistent with low pore pressure ratios (${\lambda }^{*}\approx$0–0.4). We note that some porosity estimates may imply unlikely negative ${\lambda }^{*}$ values, which may be an artifact of our calculation. Our pore pressure calculation assumes that porosity is controlled by sediment compaction. However, the material along the plate interface may not be solely subducting sediments and could contain material of igneous origin (e.g., igneous crust), which has a lower, inherent porosity not governed by a sediment compacting model. In addition, sediments likely endure shear deformation distinct from vertical compaction, which is challenging to constrain with our data and beyond the scope of our study. Overall, despite uncertainties in thermal gradient and cementation exponent (“Methods”), our results indicate that the shallow plate interface in this region is characterized by limited porosity and low pore pressure, with maximum porosity not exceeding about 20% at ~10 km landward of the trench.

Beyond approximately 17 km landward of the trench, our porosity estimates drop below 11%, which corresponds to the average porosity of the uppermost oceanic crust of the incoming plate in this region^15^. Considering that the abundant trench parallel bending faults in the incoming plate of the Shumagin Gap^2,13^ may facilitate fluid penetration into the incoming plate, we interpret the porosity farther downdip as being associated with a broader zone that encompasses the base of the upper plate, the plate interface fault zone, and the extrusive oceanic crust of the down-going plate. Beyond 45 km from the trench, where the plate interface reaches depths of approximately 10 km below the seafloor, the sensitivity of the CSEM data to deeper structures diminishes (“Methods”).

## Discussion

### Fluids and megathrust locking in the Shumagin Gap

Fluids play a critical role in regulating slip behavior within subduction zones, such as by influencing pore pressure, as elevated pore pressure can decrease effective normal stress and promote creep^1^. Our electrical resistivity model (Fig. 2) provides more direct constraints on fluid distribution in the Shumagin Gap, offering valuable insights into fluid-related mechanisms underlying subduction zone slip behaviors.

Our porosity estimate (Fig. 3) reveals relatively low porosity and a non-uniform fluid distribution along the shallow plate interface, which suggests an absence of substantial fluid accumulation and correspondingly low fluid pressures in this region. The electrical resistivity model (Fig. 2) further indicates that an along-slab conductive zone (C1) appears to diminish when it reaches the location of two seaward dipping conductive zones (e.g., C2), where fluids may drain from the plate interface and escape via permeable conduits through the upper plate. This interpretation aligns well with the collocated 2D MT study^18^, which identifies a fluid-poor segment between approximately 60 km and 90 km from the trench. This resistive segment also coincides with a high Vp velocity near the plate interface, interpreted as a potential Tertiary intrusion into the younger Chugach and Prince William Terranes^30^. This portion of the interface may inhibit updip rupture propagation, potentially confining the July 2020 M7.8 event to depths greater than 25 km within the Shumagin Gap^11,19,23^.

**Fig. 4:**
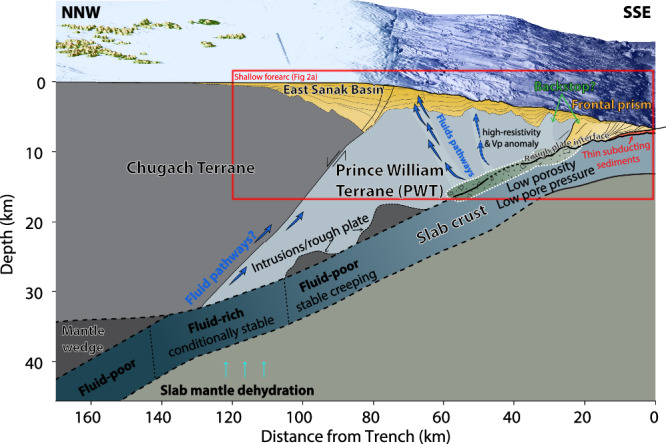
Schematic diagram illustrating a heterogeneous forearc and a fluid-poor shallow megathrust with low fluid pressure in the Shumagin Gap. The red box outlines the schematic interpretation derived from the electrical resistivity model in Fig. 2a. The shallow megathrust is characterized by thin and irregular subducted sediments and a rugged plate interface. The interface is depicted as a diffuse fractured zone, incorporating the base of the overriding plate and the uppermost downgoing oceanic crust, where dipping conductive fault zones (blue arrows) likely facilitate upward fluid migration. The observed fluid-starved distribution and inferred low fluid pressures indicate that elevated pore pressure cannot explain the low geodetic locking in this region. Instead, the structural complexity highlighted by the pronounced electrical resistivity heterogeneity both along the plate interface and within the overriding plate suggests that the intricate interplay among fluids, geological structures, and dynamic processes controls the region’s seismogenic behavior. Structural interpretations outside the red box are based on Cordell et al.^18^.

Our findings of a fluid-poor shallow plate interface, when viewed alongside recent seismic tomography results, provide a more nuanced understanding of the megathrust creep in the Shumagin Gap. Using local earthquake tomography, Wang et al.^5^ imaged broad-scale elevated Vp/Vs ratios interpreted as abundant fluids throughout the forearc in the Shumagin Gap. However, the MT, CSEM, and active-source seismic imaging results, which provide higher spatial resolution relative to regional earthquake tomography models, indicate a lack of fluids and low pore pressures along the plate interface at depths shallower than 25 km. Furthermore, while the MT model^18^ shows a conductive segment at depths >25 km (attributed to slab mantle fluids), it also reveals resistive patches farther downdip (Figs. 4 and S3), implying that fluid distribution is heterogeneous and discontinuous rather than pervasive across the entire seismogenic zone. The noted discrepancies between a seismic profile from Wang et al.^5^ and a nearly collocated MT profile^18^ may reflect considerable localized structural heterogeneity within the Shumagin Gap, which requires higher-resolution constraints (e.g., CSEM and active-source seismic imaging) to delineate detailed forearc structure and fluid distribution along the plate interface. Our inference of a fluid-poor interface is consistent with observations of thin and faulted sedimentary sections being subducted in the Shumagin Gap^3,14^ as well as with the high-velocity plate interface seen in wide-angle active source seismic imaging^30^. Therefore, given the mounting evidence for limited fluid content, low fluid pressure, and the limited presence of a consolidated sedimentary layer along the shallow megathrust (<25 km depth), mechanisms based solely on pore pressure and fault stability appear insufficient to fully explain observed creep in the Shumagin Gap.

### Factors influencing megathrust locking in the Shumagin Gap

Plate boundary heterogeneity is recognized as a key factor governing the style of subduction zone fault slip. This heterogeneity is often attributed to rough incoming seafloor, including features such as seamounts and basement relief, which create a geometrically complex network of faults upon subduction^40,42–44^. Variations in lithologies, fault geometry, fluid pore pressure, and frictional properties along the megathrust lead to heterogeneous megathrust locking and a wide range of slip behaviors, from aseismic creep and slow slip events (SSEs) to coseismic slip, in many subduction zones (e.g., Barnes et al.^45^). In the Shumagin Gap, our model (Fig. 2) reveals an along-dip variation in electrical resistivity, reflecting irregularities at the shallow plate interface. This finding aligns with seismic imaging studies that have also identified pronounced roughness at shallow plate interfaces in this region^3,14^. Such heterogeneity likely contributes to the complex slip behavior observed in the Shumagin Gap.

In addition to plate boundary complexity, the observed heterogeneous overriding plate may also have notable implications for the shallow plate interface. To first order, the depth-dependent rupture behavior in subduction zones has been attributed to changes in overriding-plate rigidity^29^. Due to reduced rigidity near the trench, coseismic slip increases toward the trench axis, amplifying tsunami hazards^29,46^. Lithological variations in the overriding plate can greatly influence the transport and storage of fluids^32^, thereby shaping the spatial distribution and impact of fluid-related processes in subduction zones. At the Hikurangi margin, for example, the degree of upper plate structural heterogeneity imaged with electromagnetic data correlates with geodetic locking patterns^4,47–49^. Similarly, seismic imaging^50^ highlights the critical role of upper-plate structures in shaping the subduction geometry, hydration state, and seismic behavior at the Nankai Trough.

As summarized in the schematic diagram (Fig. 4), our electrical resistivity model (Fig. 2) reveals a high-conductivity frontal prism near the trench, matching low seismic velocities^30^ and previously identified tsunamigenic characteristics of this region that include a heterogeneous plate interface with rugged and sparsely sedimented subducted topography, a splay fault, and a relatively small and compliant prism with low-velocity, high-conductivity, and hence low rigidity materials close to the trench^14,30,36,42,51,52^. Farther downdip, the relatively resistive margin framework is cut by subvertical conductive channels, suggesting intermittent permeable zones for upward fluid migration in between relatively competent low porosity blocks landward of the narrow accretionary prism. The conductive and permeable channels in the overriding plate facilitate fluid escape, potentially reflecting an extensional upper-plate environment in the Shumagin Gap^35,53,54^. An overriding plate with a high Vp/Vs ratio was also inferred to result from fluid migration from the plate interface^5^. Farther landward beneath the Sanak sedimentary basin, the acoustic basement comprises the high-resistivity Cretaceous Chugach Terrane. Such an increase in resistivity parallels the likely landward increase in rigidity^29^. The varying electrical resistivity from trench to outer shelf may imply substantial along-dip lithological variation of the plate interface. In addition, fault roughness along the plate boundary, lithological variability, and heterogeneous stress conditions^42^ likely contribute to the megathrust’s seismogenic complexity, particularly where rugged subducting topography interacts with fluids. The role of fluids in subduction dynamics is inherently linked to system complexity, including spatiotemporal heterogeneity in fluid sources, supply, retention, and release, and deformation-permeability interactions.

Seismic imaging studies in the Shumagin Gap observe a more hydrated subducting plate^2^ that stores abundant free water in the uppermost oceanic crust^15^. Interestingly, we observe a broad, moderately electrically conductive zone (C1, Fig. 2a) at the base of the upper plate, with porosity values comparable to those of the uppermost oceanic crust in the Shumagin Gap^15^. It is unclear whether C1 represents a zone of deformation, as collocated seismic imaging^3,14^ does not reveal a clearly correlated reflection pattern. Regardless, our resistivity model and porosity estimates suggest a relatively fluid-starved shallow interface. Therefore, beyond the frontal prism landward of the trench, the porosities surrounding the plate interface are unlikely to be solely attributed to a thin, compacted sedimentary layer. Instead, based on the general geometry of C1, we suggest that the plate interface here may be diffuse, involving part of the overriding plate and the down-going uppermost oceanic crust (Fig. 4). Due to the lack of data on the incoming plate and the limited detectability of downdip features by solely using high-frequency CSEM data, it is difficult to fully determine the possible sources of the fluids and their corresponding potential influences on the megathrust locking state at depths greater than 10 km below seafloor in this region. Nevertheless, the observed seaward-dipping fault zone (C2) might offer clues regarding the fate of fluids at the shallow plate interface in the Shumagin Gap.

The diffuse and starved fluid distribution observed at the plate interface, along with the inferred low fluid pressures, indicates that elevated fluid pressures cannot explain the low geodetic locking in this region. Instead, the pronounced electrical resistivity heterogeneity both along the plate interface and within the overriding plate highlights the structural complexity and its relationship to seismogenesis, suggesting that a more intricate interplay among fluids, geological structures, and dynamic processes may be behind the seemingly contradictory observations of this forearc system. A comprehensive explanation of megathrust locking state in the Shumagin Gap thus requires considering more than just pore-pressure and fault-stability hypotheses associated with fluids. We emphasize that while fluids play a fundamental role in subduction zone slip behaviors, variations in, for example, fluid distribution and migration, local geological structures, stress conditions, and frictional properties conspire to produce complex and segmented fault ruptures.

## Methods

### CSEM data collection

As part of the ElectroMagnetic Alaskan GeoPRISMS Experiment (EMAGE), we collected CSEM data along a profile in the Shumagin Gap (Fig. 1). This profile extends from the shelf to the outer forearc and is co-located with the ALEUT active-source seismic profile Line 5^2^. Ocean-bottom electromagnetic (OBEM) receivers were deployed at 4 km intervals along the profile. In addition, a local array of receivers was deployed on the forearc slope for 3D imaging, which is beyond the scope of this study. Each OBEM receiver was equipped with electrodes and induction coil magnetometers to measure horizontal electric and magnetic field time series in two orthogonal directions with a 62.5 Hz sampling rate. These broadband OBEM receivers can simultaneously capture both high-frequency CSEM and longer-period MT signals^55^.

For CSEM data acquisition, we deep-towed a transmitter^55^ that injected a 250–300 A source into the water column through a 293 m long horizontal electric dipole antenna. The electric current was generated in a doubly symmetric waveform with a 0.25 Hz fundamental frequency^56^, which provides higher signal-to-noise ratios for higher frequencies, allowing for a wide frequency spectrum to better constrain the crustal resistivity structure. During deep-tow operations, the transmitter was maintained at an approximate altitude of 100 m above the seafloor (measured by an acoustic altimeter) to ensure energy coupling with the seafloor while avoiding rugged topography. On the continental shelf section (>83 km from trench) of the profile, where water depths are less than 100 m, the transmitter was towed a few meters below the surface approximately 4 m astern of the vessel. Seawater conductivity, temperature, depth and sound velocity data were measured by sensors attached to the transmitter throughout the operation, as these parameters are critical for navigation accuracy and CSEM inversion. With the exception of the OBEM receivers deployed on the shelf, whose positions were estimated to be the same as their drop locations, the positions of OBEM receivers were determined using classical long-baseline acoustic ranging, while the transmitter positions were determined using an inverted long-baseline acoustic navigation system^57^.

### CSEM data processing

We apply a robust stacking method^56^ to enhance the signal-to-noise ratio (SNR) across multiple frequencies and estimate the variance for the data. First, the raw CSEM time series data were segmented into 4-s windows, corresponding to the fundamental period of the transmitted waveform. The segmented data were transformed into frequency-domain transfer function (TF) estimates of the Earth response by pre-whitening, Fourier transformation, and post-darkening. After normalization by the source dipole moment, the short window (4 s) TF estimates were robustly stacked in 120 s intervals to increase the SNR. After processing, the TF estimates are converted into amplitude and phase responses for each receiver as a function of transmitter-receiver offset at each frequency. The variance was calculated for each stacked data point from residuals after removing linear trends, providing an estimate of the data error. The navigational information of the transmitter and receivers was merged with the stacked amplitude and phase responses. Note that accurate navigation is critical for CSEM inversion and represents the largest source of error. To account for this, we apply a 3% error floor to the data.

For our inversion, we used CSEM data at the first, third, and seventh waveform harmonics (0.25, 0.75, and 1.75 Hz), which provide peak energy, i.e., the highest sensitivity to the depth range of interest while maintaining a good SNR. We omitted the data with an SNR below 2 and those with transmitter-receiver offsets $\le$ 2 km due to navigation uncertainties^58^. We removed any obvious outliers after stacking. Example data responses from site 540b on the shelf (approximately 106 km landward of the trench) and site 533 on the forearc slope (approximately 34 km landward of the trench) are presented in Fig. S4.

### CSEM inversion and mesh design

We applied the 2D inversion code MARE2DEM^59^ to image the 2D electrical conductivity structure from the CSEM data. MARE2DEM does not require prior knowledge about the model, so we set a 10 $\Omega$ m uniform half-space as the initial model for the inversion. The core volume of particular interest starts from the trench to approximately 120 km landward along the profile, extending to about 18 km below the seafloor. The quadrilateral cells at the surface are about 50 m height $\times$ 150 m width, with height following growth factor of 1.05 to about 10 km below the seafloor. For depths between ~10 km and ~18 km, a coarser horizontal resolution of 300 m per cell was applied while maintaining the same vertical growth factor. High-resolution bathymetry recorded by deep-towed SUESI was incorporated in the core area of interest in our model. The mesh was refined beneath the CSEM receivers. To avoid the boundary effect, a triangle-meshed outer padding region extends from −500 km to 600 km relative to the trench axis, from 100 km above sea level and to a depth of 1000 km, with the largest possible triangles to keep computation efficiency. Resistivity values for the air and seawater were determined and subsequently held fixed during the inversion. A seawater resistivity model was discretized using near-seafloor conductivity measurements from the CSEM transmitter and background climatological data from WOA18 (Northern Hemisphere summer, 2005–2017 average). The air was assigned a resistivity of 10^13^ $\Omega$ m.

Our preferred model (Fig. 2) converged to a root mean square (RMS) misfit of 0.9025. Fig. S5 summarizes the CSEM data and response matrices for each frequency from all receivers. Another detailed misfit distribution is provided in Fig. S6. The elevated misfits near ~62 km and ~50 km landward of the trench axis are likely attributed to rapid bathymetry changes and rapid transmitter altitude variations during re-diving, respectively. As inline data is primarily sensitive to the vertical resistivity^60^, we present the vertical resistivity in Fig. 2, while horizontal resistivity and the anisotropy ratio are shown in Fig. S7. The inherent anisotropy appears to be minor, as demonstrated by the isotropic inversion (Fig. S8), which can achieve a similar RMS misfit even with fewer degrees of freedom and recover essentially the same first-order conductivity structures as the anisotropic inversion. Variations in the anisotropy penalty weight introduce only marginal changes to the model, and the anisotropy ratios remain near zero except near the surface (Fig. S7). These suggest that strong anisotropy is not required to explain the data. We choose the vertical resistivity from the anisotropic model because it converges to a similar target RMS misfit more rapidly and robustly without sacrificing model smoothness, providing better near-surface fits. Note that OBEM sites on the shelf were not precisely located in a shallow water environment, with drop locations used as site positions. In addition, the error floor might affect the convergence and the imaging of the shallow structures, especially for the northernmost data points on the shelf.

### Resolution and sensitivity tests of forearc conductors

We first tested whether our CSEM data can resolve extending C1 farther downdip. Starting from our inverted vertical electrical resistivity model (Figs. 2 and S1), we generated synthetic data by directly introducing a thick conductive layer (5 $\Omega$ m) along the plate interface, extending downdip to 20 km depth (Fig. S9a). Assuming the same uncertainty distribution as the observed real data, the inversion of this synthetic dataset converged to a misfit of 0.9047. However, we cannot identify clear anomalies attributable to the added structure; as depth increases, the data sensitivity diminishes. Although the introduced structure causes measurable RMS changes up to 60 km landward of the trench, this effect is generated by a substantial 80% decrease in resistivity, far greater than the 10–20% changes typically used in our sensitivity tests. Overall, our CSEM data have sufficient sensitivity to resolve a conductive feature along the plate interface up to ~45 km landward of the trench (~10 km below seafloor), but lose resolution farther downdip. Although our image (Fig. 2) extends to 17 km below sea-level, the data have only limited sensitivity and insufficient resolution at this depth. Therefore, using only CSEM data, we cannot definitively determine whether a conductive structure exists farther downdip along the plate interface in the Shumagin Gap. However, collocated MT data^18^ are sensitive to the downdip portion of the plate interface and image an electrically resistive segment (Fig. S3). This indicates a lack of fluids along the plate interface between approximately 50 km and 90 km from the trench. We speculate that C2 (Fig. 2) may facilitate sufficient fluid drainage from the plate interface to render it fluid-poor.

We also assessed whether our data can resolve features beneath the Sanak Basin (C3), where a deep-rooted, large normal fault is identified by collocated seismic studies^14,30^. Beneath the Sanak Basin, we add a conductive anomaly along the fault, extending from approximately 5 km to 20 km depth (Fig. S9a). Following the same strategy as mentioned above, the inversion converged with a misfit of 0.9045. Yet again, the inverse model does not exhibit distinct anomalies corresponding to the introduced structure, indicating that we are unable to resolve a conductor beneath the basin. Given the presence of the thick Sanak sedimentary basin, it is reasonable that our data lack the sensitivity required to resolve deeper structures such as conductive fault zones. It is noteworthy that we do not expect a similar loss of sensitivity in the conductive frontal prism because it is thinner, and the target of interest—the plate interface—is shallower (<3.5 km below the seafloor) compared to the deep conductor tested beneath the Sanak Basin (>5 km). While transmitter coverage is limited near the trench (effective coverage starts at ~18 km landward), the high SNR of our CSEM receiver data ensures that the data retain sufficient resolution to estimate porosity along the plate interface from approximately 10 to 20 km landward of the trench.

In our model (Fig. 2), C1 and C2 represent two weak conductive structures. C1 exhibits resistivity around 10 to 40 $\Omega$ m, and C2 ranges from approximately 30 to 55 $\Omega$ m. To evaluate the data sensitivity to these structures, we increased their resistivity values by 10 $\Omega$ m and forward modelled the data responses. Because our CSEM data lack sufficient sensitivity to the plate interface below 13 km depth or beyond 45 km landward of the trench, we only use the shallower, more reliable portion of C1 in this sensitivity test. The approximate geometry of C2 is shown in Fig. S9a as well. Both C1 (Fig. S9c) and C2 (Fig. S9b) show clear local RMS changes, demonstrating that, despite the inherent differences in resistivity and depth, our CSEM data display sufficient sensitivity to perturbations in both C1 and C2.

### Porosity estimation

To estimate the porosity from our preferred electrical resistivity model (Fig. 2), we apply the empirical Archie’s law^38^:

1 $$\phi={\left(\frac{{{{{\rm{\rho }}}}}_{f}}{{{{\rm{\rho }}}}}\right)}^{\frac{1}{m}}$$

where $\phi$ is the porosity, $\rho$ is the bulk resistivity, ${\rho }_{f}$ is the estimated pore fluid resistivity, and $m$ is the cementation exponent. Since direct salinity measurements are unavailable and the potential impact of pore fluid freshening on porosity estimates is considered minor^37^, we assume the pore fluid has a seawater salinity of 3.5 wt%, where the fluid resistivity ${\rho }_{f}$ depends on temperature. For lower-temperature regions ($T$ < 200 °C), we use a cubic relationship to calculate the pore fluid resistivity^61^:

2 $${\rho }_{f}\left(T\right)={\left[2.903916(1+2.97175\times {10}^{-2}T+1.5551\times {10}^{-4}{T}^{2}-6.7\times {10}^{-7}{T}^{3})\right]}^{-1}$$

valid for temperatures between 0 °C and 200 °C. At higher temperatures, possibly considered in our analyses, we smoothly interpolate the pore fluid resistivity by applying an empirical relationship^62^, which is also depth (pressure) dependent.

We assume a linear geothermal gradient with depth and set a geothermal structure of the plate interface following

3 $$T={T}_{0}+G\Delta z$$

where ${T}_{0}$ is the temperature at the seafloor, which is extrapolated from the temperature data recorded by the CSEM transmitter, $G$ is an assumed geothermal gradient, and $\Delta z$ is the depth below the seafloor. In our baseline case, we use a constant geothermal gradient of *G* = 10 °C km^−1^ and adopt a cementation exponent in Archie’s law of $m=2$ (corresponding porosity model is shown in Fig. S2).

We employ this simplified geothermal gradient assumption primarily due to the considerable uncertainties inherent in thermal modeling of shallow forearc subduction zones. Unlike deeper regions, where slab geometry and geodynamic modeling help constrain the thermal structure, the shallow forearc in this region lacks heat flow data on the seafloor, which hinders a clear understanding of the shallow (upper tens of kilometers) thermal structure near the trench. While the average geothermal gradient from a reference thermal model^63^ is less than 10 °C km^−1^ for shallow subduction zones, the accuracy of this estimate is not well constrained. For example, the potential influence of shear heating and fluid circulation in the subducting crust^64–66^ suggests that subduction zones can be warmer than predicted in thermal models that ignore these effects. Complex shallow forearc thermal conditions also make this question more complicated. Furthermore, while more complex thermal models are available in other regions, applying them to this data-limited, shallow region requires local heat flow observations and would likely yield only a minor impact on our porosity estimates because we attempt to account for these uncertainties within our bounds. Indeed, our comparative calculation using the plate interface temperature from the van Keken and Wilson^67^ model reveals only a minor effect on the resulting porosity trends (Fig. 3). Our simplified approach for calculating the plate interface thermal structure, therefore, allows for greater transparency regarding its assumptions and limitations. To explore end-member scenarios and take account of the uncertainties, we vary the thermal gradient from *G* = 5 °C km^−1^ up to *G* = 15 °C km^−1^ and vary $m$ between 1.8 and 2.2 to represent differences in pore geometry. We note that our porosity estimates likely represent a conservative upper bound. The presence of clay minerals (e.g., smectite) would enhance bulk conductivity via surface conduction, while the presence of fracture porosity would imply a lower cementation exponent ($m < 2$). Accounting for either of these factors would result in porosity estimates even lower than those reported here. Thus, the potential influence of clays or fractures reinforces our conclusion of a low-porosity, fluid-poor plate interface.

### From porosity to fluid pressure

We present fluid pressure using the modified pore pressure ratio, ${\lambda }^{*}=\left({P}_{f}-{P}_{h}\right)/\left({\sigma }_{v}-{P}_{h}\right)$, where ${\sigma }_{v}$ is the total vertical stress, ${P}_{h}$ is the hydrostatic pressure, and ${P}_{f}$ is the pore fluid pressure. We assume hydrostatic pore fluid pressure for normally consolidated sediments on the incoming oceanic plate. Since the pore fluid pressure is defined by ${P}_{f}={\sigma }_{v}-{\sigma }_{v}^{{\prime} }$, where ${\sigma }_{v}^{{\prime} }$ is the vertical effective stress, we can rearrange the expression of ${\lambda }^{*}$ to solve for the vertical effective stress as ${\sigma }_{v}^{{\prime} }=\left(1-{\lambda }^{*}\right)\left({\sigma }_{v}-{P}_{h}\right)$. The total vertical stress ${\sigma }_{v}$ is calculated by integrating the bulk density downward, which is estimated from our porosity structure, assuming a grain density of 2700 kg m^−3^. The hydrostatic pressure ${P}_{h}$ is calculated along the plate interface, assuming a fluid density of 1030 kg m^−3^. By using a compression trend that links void ratio (and thus porosity) and vertical effective stress^68,69^ and the specific parameters (compression index and void ratio projected at zero effective stress) reported by Li et al.^3^ for our study region, we can predict the porosity along the plate interface as a function of distance landward of the trench for a given presumed pore pressure ratio ${\lambda }^{*}$.

## Supplementary information

Supplementary Information File

Transparent Peer Review File

## Data Availability

Raw EM time-series data and relevant metadata are available at 10.60521/331594. The processed CSEM data and the inversion model files presented in Fig. 2 are available at 10.5281/zenodo.15400332. The seismic reflection data shown in Fig. 2b, c and Fig. S1 are available at https://www.marine-geo.org/tools/entry/MGL1110. The World Ocean Atlas (WOA) is publicly available at https://www.nodc.noaa.gov/OC5/woa18/. Correspondence and requests for materials should be addressed to Yinchu Li.

## Code availability

CSEM data were inverted with MARE2DEM, which is available at https://mare2dem.bitbucket.io.
