# Supplementary material for: Electromagnetic imaging reveals insufficient fluids to explain shallow megathrust creep at the Shumagin Gap
Source: Nat Commun. 2026 Mar 28;17:5356. doi: 10.1038/s41467-026-71176-7 (PMC13276360; doi:10.1038/s41467-026-71176-7)
Supplement: Supplementary file 1 — Supplementary Information File [file 41467_2026_71176_MOESM1_ESM.pdf]

Supplementary Materials for

Electromagnetic imaging reveals insufficient fluids to explain shallow megathrust creep at the Shumagin Gap

Authors:

Yinchu Li[1]*, Samer Naif[1], Kerry Key[2], Steven Constable[3], Rob L. Evans[4], Donna J. Shillington[5], Anne Bécel[6], Darcy Cordell[7,8]

Affiliations:

[1]School of Earth and Atmospheric Sciences, Georgia Institute of Technology, Atlanta, GA, USA.

[2]Deep Blue Geophysics, LLC, Los Angeles, CA, USA.

[3]Scripps Institution of Oceanography, La Jolla, CA, USA.

[4]Department of Geology and Geophysics, Woods Hole Oceanographic Institution, Woods Hole, MA, USA.

[5]School of Earth and Sustainability, Northern Arizona University, Flagstaff, AZ, USA.

[6]Lamont-Doherty Earth Observatory, Columbia University, Palisades, NY, USA.

[7]Centre for Science, Athabasca University, Athabasca, Alberta, Canada.

[8]Department of Physics, University of Alberta, Edmonton, Alberta, Canada.

*Corresponding author. Email: yli3354@gatech.edu

This file includes:

Supplementary Figs S1 to S9

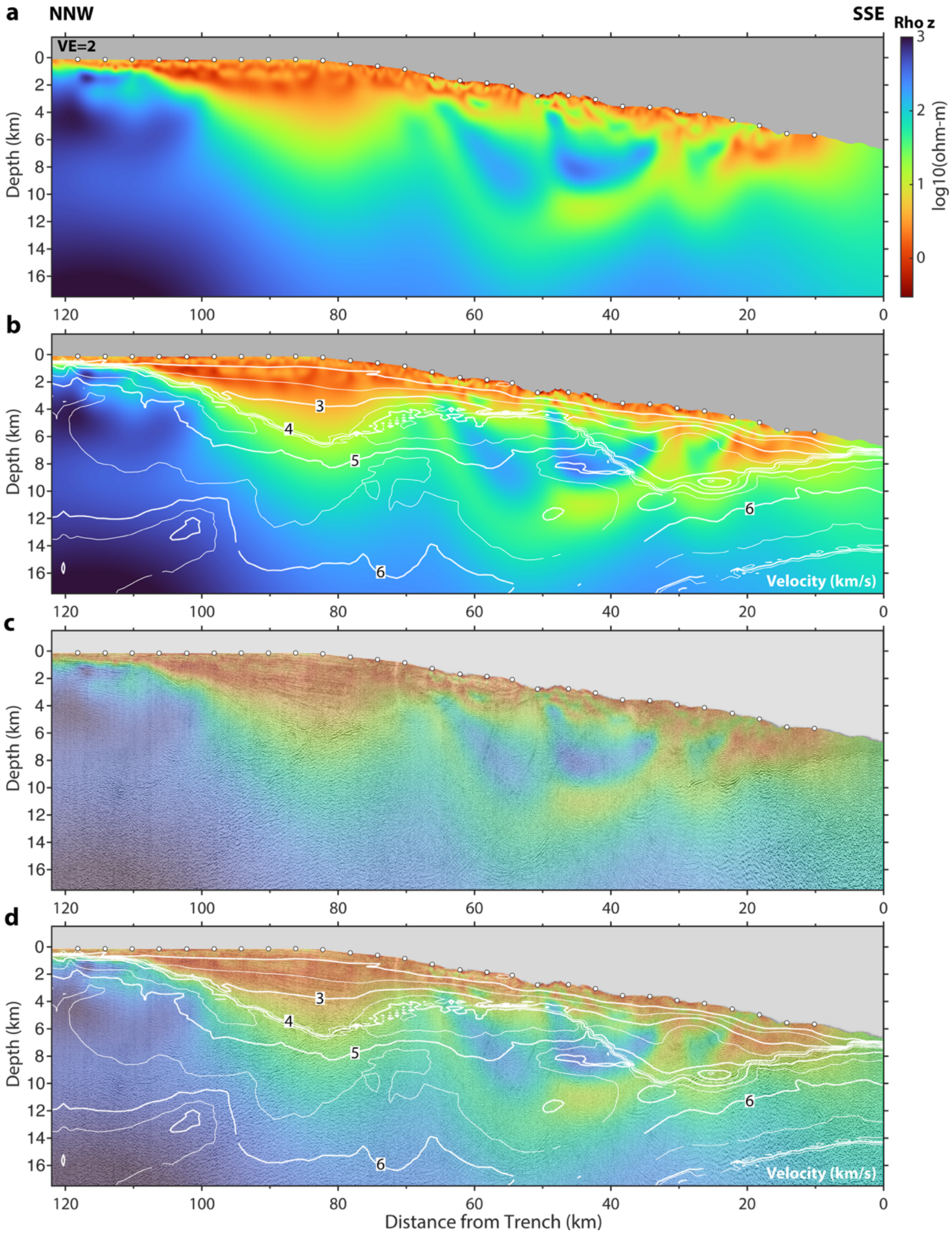

Fig. S1. Comparison of uninterpreted resistivity model, seismic, and P-wave velocity model. (**a**) Same image shown in Fig. 2 without interpretations. (**b**) Resistivity model with collocated P-wave velocity[1]. (**c**) Resistivity model with collocated seismic result[2]. (**d**) Resistivity model with collocated seismic result[2] and P-wave velocity[1].

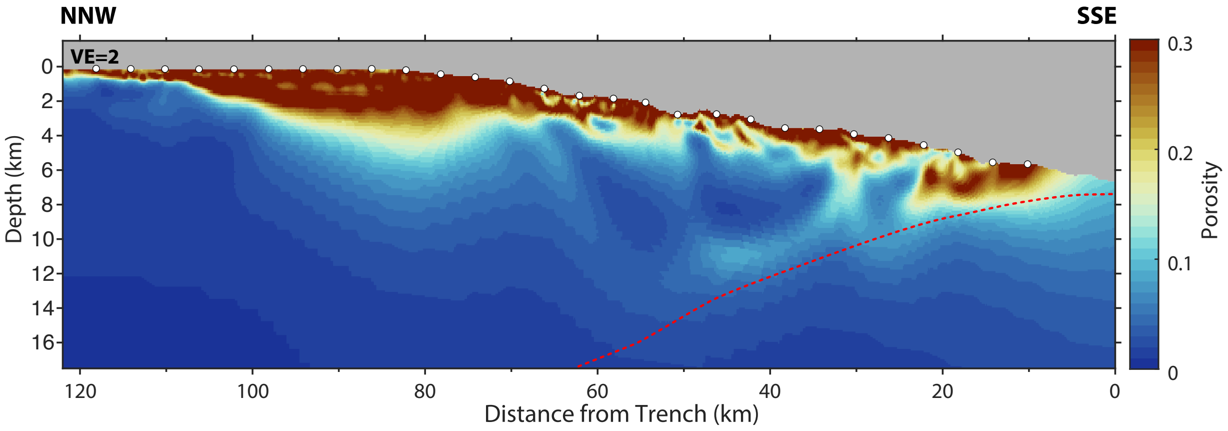

Fig. S2. Porosity cross section. The red dashed line represents the plate interface used in this study and along which porosity is estimated in Fig. 3.

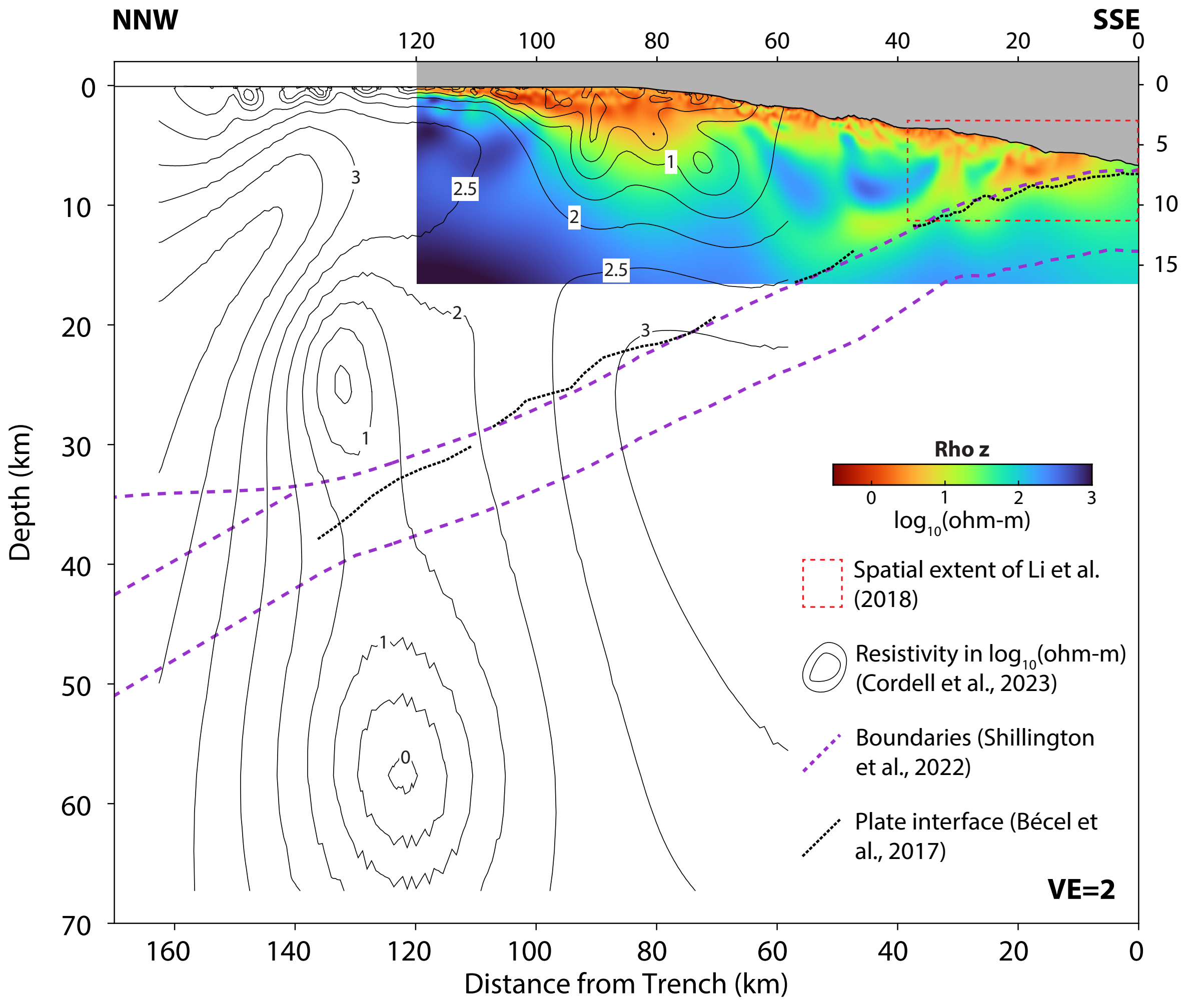

Fig. S3. Comparison between our preferred resistivity model and the MT result[3]. Background model is our preferred resistivity model shown in Fig. 2 and Fig. S1. Black solid lines are the contour line at 0.5 log unit intervals from the resistivity model from[3]. Red dashed rectangle outlines the spatial extent of P-wave velocity model from[4]. Black dashed line indicates the plate interface from[2]. Purple dashed lines denote slab crust boundaries from Shillington et al.[1]. The uppermost segment located at >135 km represents the overriding plate Moho.

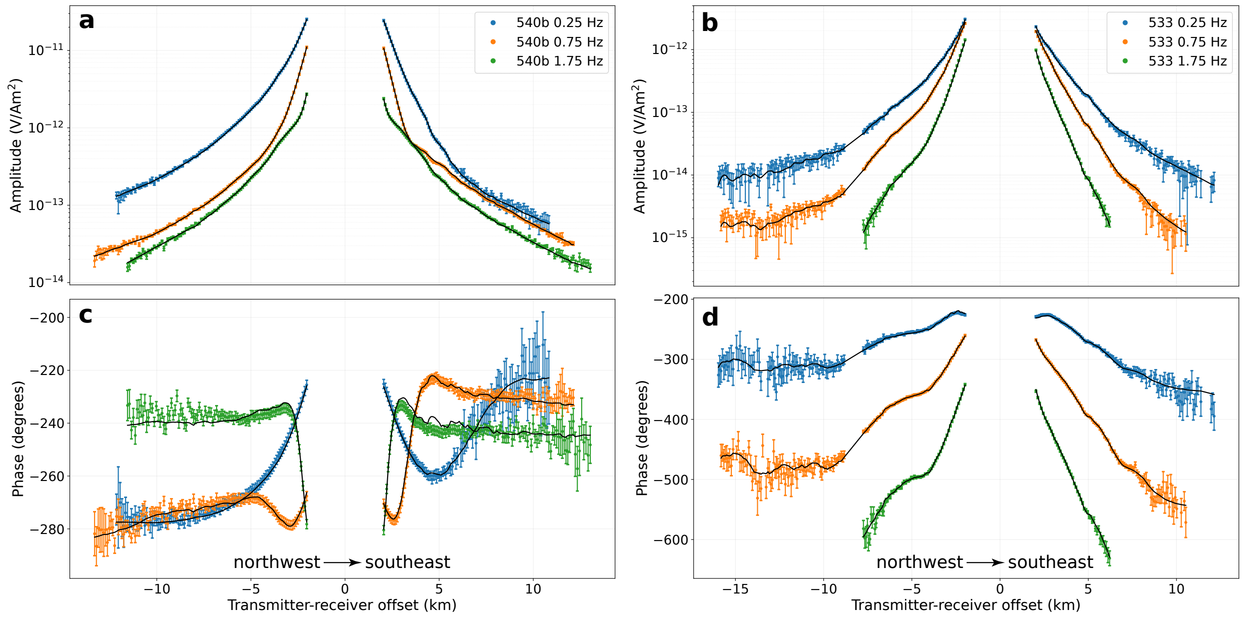

Fig. S4. Example CSEM amplitude and phase responses. Amplitude (**a**, **b**) and phase (**c**, **d**) at 0.25, 0.75, and 1.75 Hz are displayed for station 540b (shelf) and station 533 (forearc slope). The variance of detrended residuals for each stacked data point provides the data error bar. The solid black line is the model response.

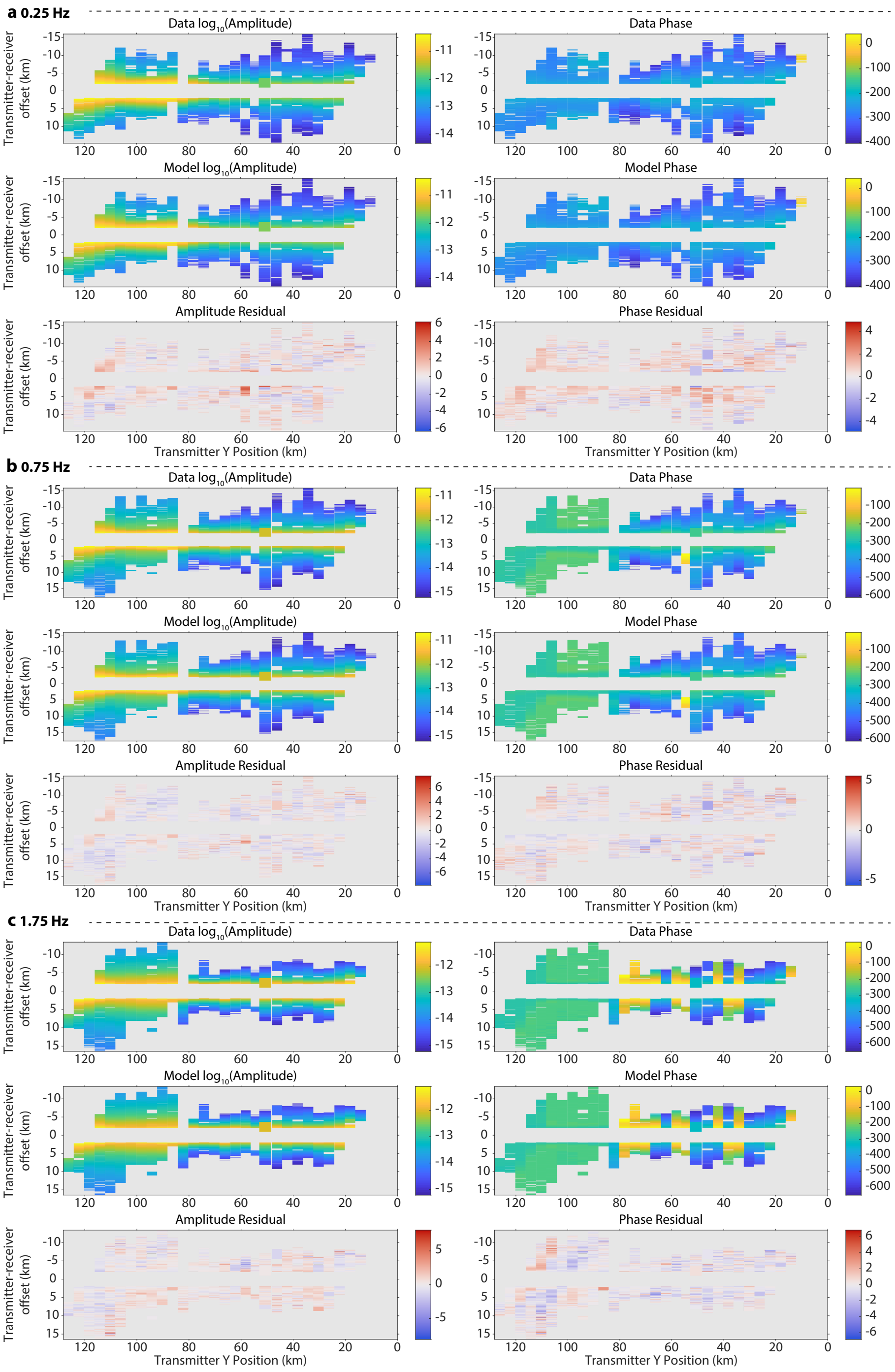

Fig. S5. CSEM data and model fit matrices. Panels **a-c** correspond to frequencies of 0.25, 0.75, and 1.75 Hz, respectively. Within each panel, data (top), model response (middle), and residual (bottom) for amplitude (left) and phase (right) are shown as a function of distance from the trench and transmitter–receiver offset.

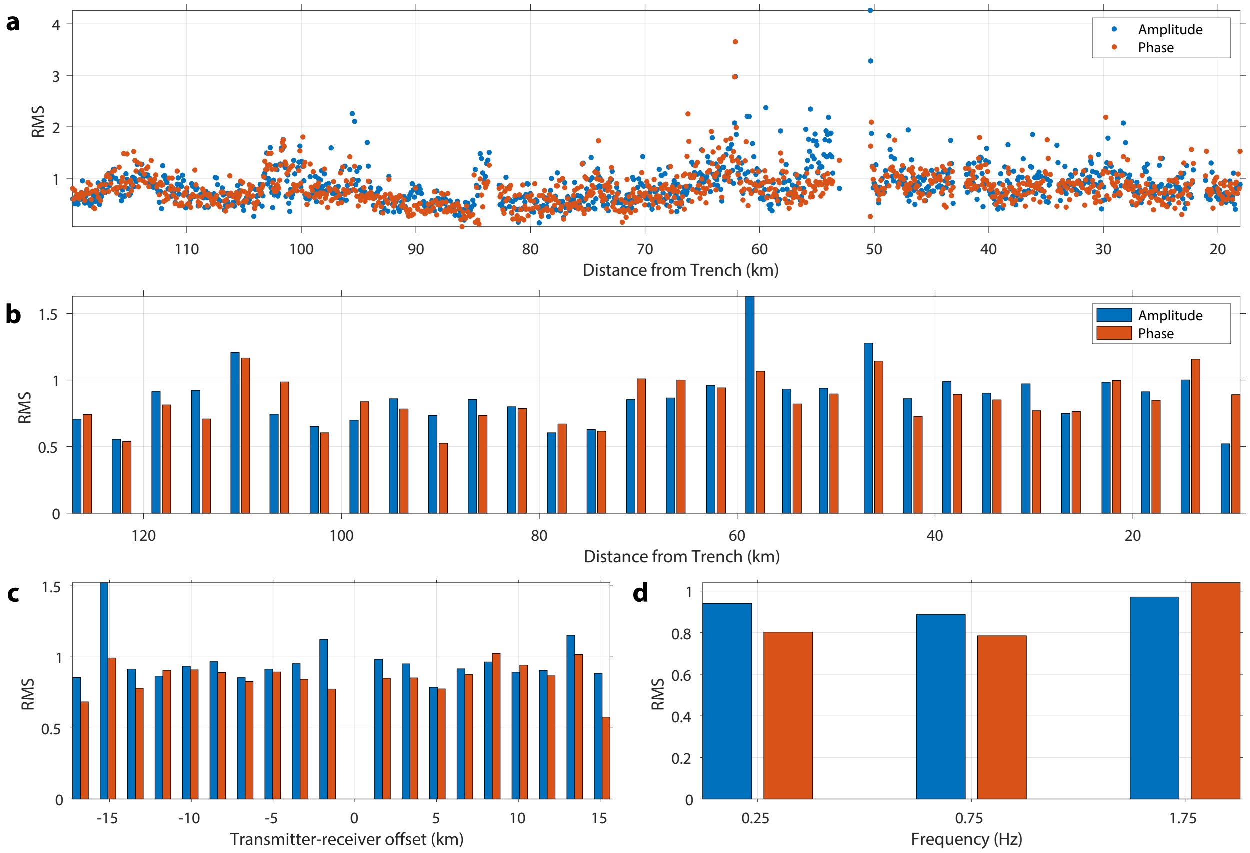

Fig. S6. Model fit breakdown. Root mean square (RMS) misfit of amplitude and phase data calculated (**a**) for given transmitter position; (**b**) for each receiver; (**c**) as a function of transmitter-receiver offset; (**d**) for each frequency.

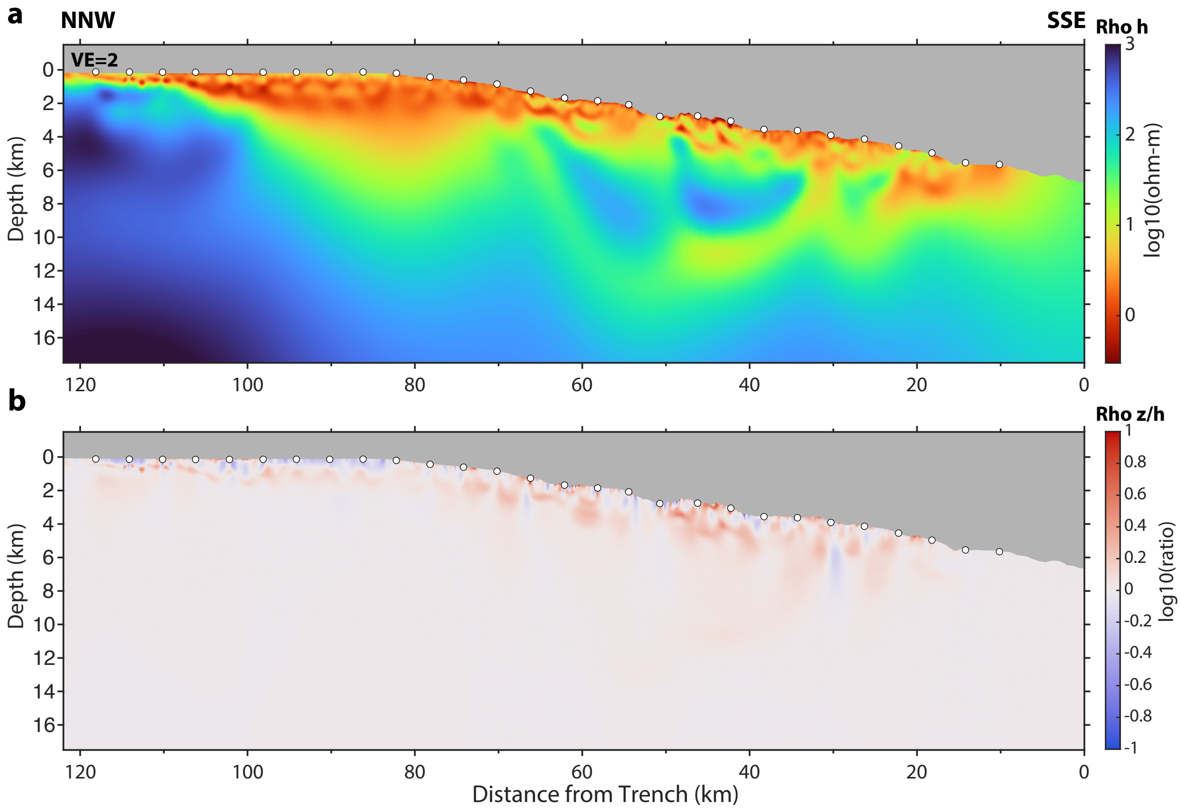

Fig. S7. Anisotropic inversion. (**A**) Horizontal resistivity model. (**B**) Anisotropy ratio between vertical and horizontal electrical resistivity.

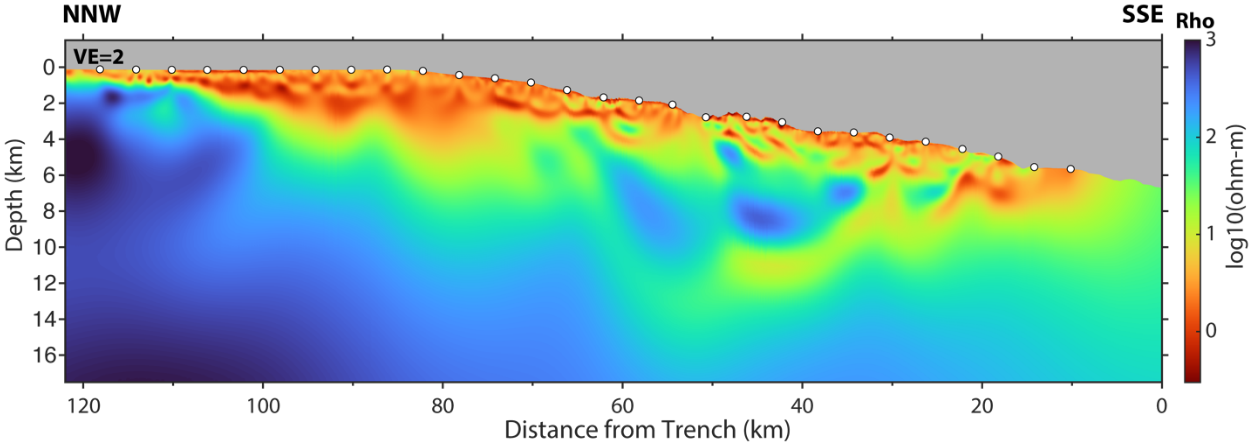

Fig. S8. Isotropic inversion for the same CSEM data set and RMS = 1. Isotropic inversion can achieve RMS = 1 and the model reproduces the first-order conductivity structure of the preferred resistivity model (Fig. 2), with only minor near-surface differences.

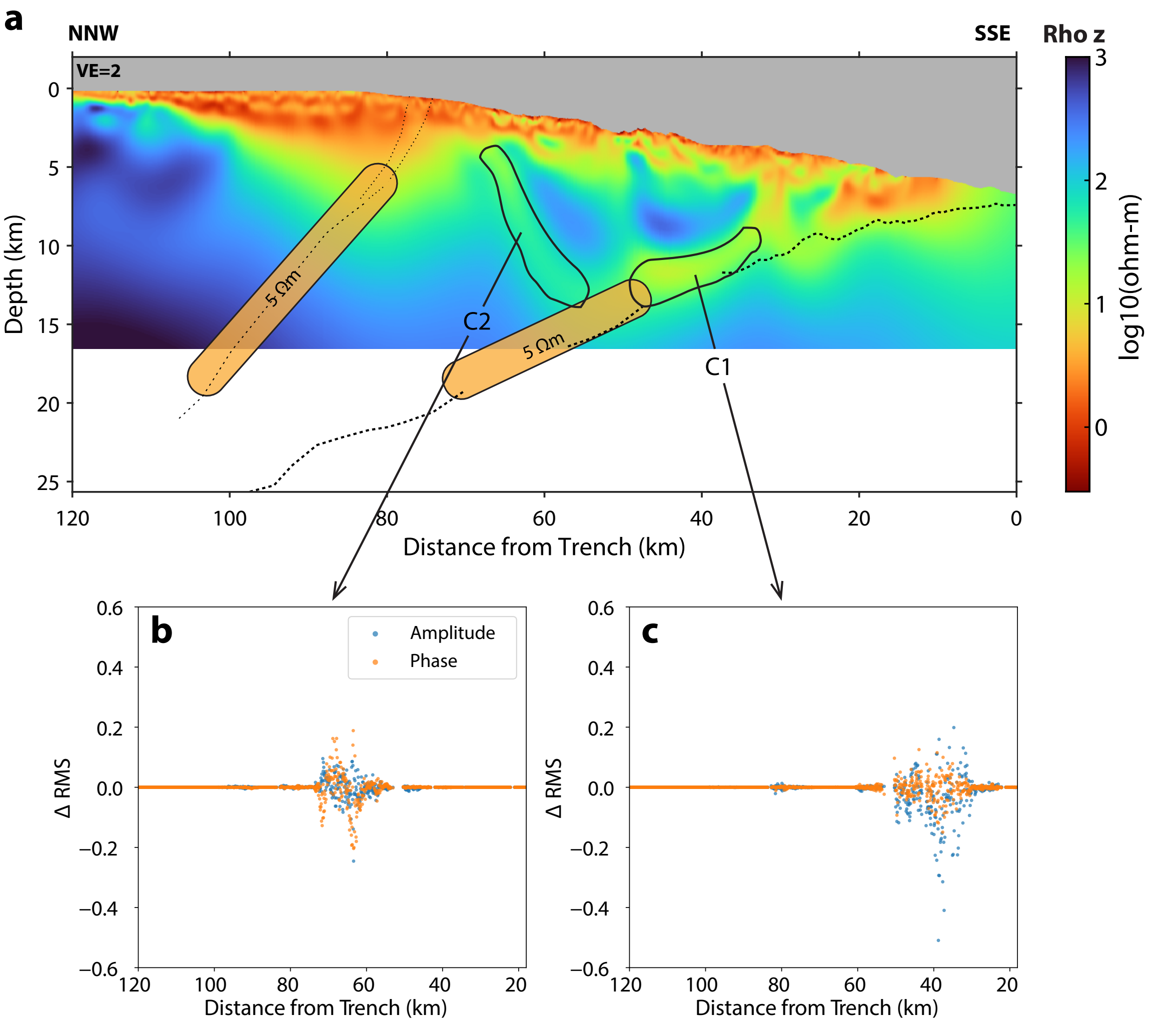

Fig. S9. Sensitivity to forearc conductors of interest. (**a**) Geometries used in the sensitivity analysis. Black polygons delineate conductors C1 and C2. Orange shaded areas represent potential conductive features (e.g., beneath the basin and farther downdip of C1) included in the sensitivity tests. (**b**, **c**) Difference in model misfit after increasing the resistivity of conductor C1 (**c**) and C2 (**b**) by 10 Ω m from the preferred model, plotted against transmitter position. Blue and orange dots denote changes in amplitude and phase misfit, respectively.

References

1. Shillington, D. J., Bécel, A. & Nedimović, M. R. Upper Plate Structure and Megathrust Properties in the Shumagin Gap Near the July 2020 M7.8 Simeonof Event. *Geophysical Research Letters* **49**, e2021GL096974 (2022).
2. Bécel, A. *et al.* Tsunamigenic structures in a creeping section of the Alaska subduction zone. *Nature Geosci* **10**, 609–613 (2017).
3. Cordell, D. *et al.* Forearc seismogenesis in a weakly coupled subduction zone influenced by slab mantle fluids. *Nat. Geosci.* **16**, 822–827 (2023).
4. Li, J. *et al.* Connections between subducted sediment, pore-fluid pressure, and earthquake behavior along the Alaska megathrust. *Geology* **46**, 299–302 (2018).
