# Supplementary material for: Electromagnetic imaging reveals insufficient fluids to explain shallow megathrust creep at the Shumagin Gap
Source: Nat Commun. 2026 Mar 28;17:5356. doi: 10.1038/s41467-026-71176-7 (PMC13276360; doi:10.1038/s41467-026-71176-7)
Supplement: Supplementary file 2 — Transparent Peer Review File [file 41467_2026_71176_MOESM2_ESM.pdf]

nature portfolio

# Peer Review File

# Electromagnetic imaging reveals insufficient fluids to explain shallow megathrust creep at the Shumagin Gap

Corresponding Author: Mr Yinchu Li

**This file contains all reviewer reports in order by version, followed by all author rebuttals in order by version.**

Version 0:

Reviewer comments:

Reviewer #1

(Remarks to the Author)
Review report NCOMMS-25-77104

The authors carried out controlled-source EM profiling along a 200km profile over one of the rupture areas of the July 2020 Mw 7.8 earthquake in the Shumagin Gap, to clarify shallow slip behaviour and the upper boundary in terms of fluid distribution. The authors successfully imaged the resistivity section in the near-trench forearc region to a depth of 16km, revealing trench-normal heterogeneity in the prism and insufficient fluid to explain the weak plate coupling.

The topic is interesting, and the objective is clear. The measurement methodology and inversion schemes are state-of-the-art, and the resistivity model is robust.

The Shumagin Gap is known to have a weakly coupled interface from geodesy, but the fluid distribution at the Gap has been debated in seismology and magnetotellurics. The present paper reports that the near-trench sedimentary fluid supply does not extend to greater depths within the plate interface. This conclusion is essential and casts new questions about the weakly coupled plate interface to the Shumagin Gap.

The reviewer has the following comments.
Regarding the influencing factor in plate coupling, fluid is a candidate. However, the existence of a seamount or the topography (plate-bending geometry) of the downgoing plate is another. Can the authors say whether the Shumagin Gap lacks seamounts or has a smoother plate geometry than the surroundings?
Is there any other unique geophysical nature (excluding the geodesy) of the Shumagin Gap, as compared with the surrounding past-rapture area (M9 class)? To this reviewer, the Shumagin Gap is not as earthquake-prone as the surrounding areas, as it has had multiple M7-class earthquakes, as shown in Figure 1.
As trench-normal heterogeneity exists, the deeper part of the plate (where M7-class ruptures occurred) may contain fluids and have triggered the rupture. Will this idea conflict with the previous MT result?
The concluding figure, together with the previous magnetotelluric results, should emphasize the conclusion.
In the future, another CSEM profile over the surrounding locked segments (e.g., Semidi) will directly clarify the authors' argument for water-starved subduction at the Shumagin Gap. Instead, the supply of fluids for the "locked" segment surrounding the Shumagin Gap may have triggered the large ruptures.
A graphical comparison or compilation of past similar studies (e.g., Nicaragua, Hikurangi) on the strongly/weakly coupled interface and the incoming sediments near the trench will strengthen the authors' argument.
Regarding the interpretation of the porosity from the resistivity, how about the influence of clay minerals, like smectite? How is the fracture porosity?

Specific comments:
Fig.1: The contour lines of the slab depth are shown with a 10km interval, but the depths should also be annotated in the figure.
Fig. 1 and L. 283: The SSEs can be mapped in Fig. 1.
Fig.3 Caption: Lambda should be defined, or the definition should be referenced.

Reviewer #2

(Remarks to the Author)
This manuscript presents a new 2-D high-resolution image of electrical resistivity in the Shumagin seismic gap, using the latest collected controlled-source electromagnetic data from the seafloor. The authors further interpret the low-resistivity anomalies as rock porosity filled with fluids. The results show that the plate interface in the top 10 km has a lower porosity than previously expected. This is inconsistent with previous suggestions that abundant fluids on the plate interface result in a weak interseismic plate coupling in the Shumagin Gap. The authors suggest that it is the interplay of the whole system, rather than simply the fluids on the plate interface, that modulates the megathrust in this region.

Since electromagnetic data analysis is not my expertise, my comments mainly focus on interpretations and defer the technical part to other reviewers.

I think this is an exciting discovery. It, together with some recent studies, challenges a long-standing simplified model that the fluids on the plate interface dominate the megathrust. I recommend a minor revision. Here are my comments, as the authors had addressed many of my early concerns.

Line 39: The hypothesis needs a reference, ideally in rock physics.

Line 59: I suggest adding "…, although the shallow portion (<10 km) of the megathrust near the trench remains poorly imaged."

Lines 260–276: These sentences need to be more precise on the depths. Earthquake tomography (Wang et al., 2024) and MT images (Cordell et al., 2023) show fluids on the interface deeper than 25 km, but CSEM (this study) and active-source seismic imaging (Li et al., 2018) show a fluid-starving interface shallower than 25 km. In contrast, interseismic plate coupling models usually extend to 45 km depth, and have very low resolution near the trench (<15 km depth). So do the authors propose that the entire megathrust weak coupling is not related to fluids on the interface or only the shallow portion of the megathrust? Please be more specific on the depths.

Reviewer #3

(Remarks to the Author)
Although I am unable to scrutinize fine technical details of the data collection and processing reported in this paper, there is enough information in the paper to help me assess the validity and importance of the scientific story. As far as I can tell, the data and the research work are of high quality, and the scientific conclusions are important. The paper thus deserves timely publication in Nature Communications with some minor revision. I am returning an annotated pdf copy of the manuscript marked with my comments. I am only reiterating a few of them here.

The title of the paper reads like that of a newspaper report. This is unfortunately what journals like Nature and Science are promoting today. I wish these journals could understand that objectivity is the lifeline of science, and an assertion or claim is inappropriate for the title of a scientific paper. I don't know what the authors can do about it given the (strangely) tight word limit imposed by these journals, but to the minimum I think readers deserve to know from the title what scientific approach is used (e.g., electromagnetic imaging, or at least geophysical imaging).

The meaning of "weak coupling" is unclear. Obviously, this paper is trying to address whether fluid controls slip behavior, creeping vs. stick-slip. A decrease in effective normal stress leads to a lower shear strength, but it does not translate to slip behavior. See Wang and Dixon (2004, https://doi.org/10.1029/2004EO180005) and Wang and Bilek (2014, ref 45 in this manuscript)). Instead of weak or strong coupling, I suggest that clear wording is used as needed such as "lower shear stress" (force), "velocity-strengthening" (property or behavior), "creeping" (kinematics), etc.

I suggest that the authors make a better effort in linking figures to text and connecting different figures. See comments in the annotated manuscript.

To me, having pore fluid pressure lower than hydrostatic is not physically unrealistic, although one can consider it "unlikely". I made some suggestion to rephrase line 222.

In the discussion of applicability of the sediment compaction model in lines 221-226, only the presence basalt is mentioned. I made a suggestion to consider sediment deformation (shear vs. in situ compaction).

Kelin Wang
November 2025

Version 1:

Reviewer comments:

Reviewer #1

(Remarks to the Author)

Review report on NCOMMS-25-77104A
The previous comments from this reviewer were answered point by point, and the manuscript was revised accordingly. The paper is publishable.

**Open Access** This Peer Review File is licensed under a Creative Commons Attribution 4.0 International License, which permits use, sharing, adaptation, distribution and reproduction in any medium or format, as long as you give appropriate credit to the original author(s) and the source, provide a link to the Creative Commons license, and indicate if changes were made.
In cases where reviewers are anonymous, credit should be given to 'Anonymous Referee' and the source.
The images or other third party material in this Peer Review File are included in the article's Creative Commons license, unless indicated otherwise in a credit line to the material. If material is not included in the article's Creative Commons license and your intended use is not permitted by statutory regulation or exceeds the permitted use, you will need to obtain permission directly from the copyright holder.
To view a copy of this license, visit https://creativecommons.org/licenses/by/4.0/

Response to Reviewers' comments on *Nature Communications* manuscript NCOMMS-25-77104

"Insufficient fluids to explain weak plate coupling along the shallow megathrust at the Shumagin Gap" by Li et al.

Reviewers' comments are in **dark gray** and our responses are in **dark blue**. All line numbers in our responses refer to the revised manuscript (clean version). We have also made some edits to meet the *Nature Communications* format requirements. The supplementary information file has been updated accordingly.

We thank the reviewers for their insightful comments which we believe have significantly improved the clarity and robustness of the manuscript.

## Reviewer #1

The authors carried out controlled-source EM profiling along a 200km profile over one of the rupture areas of the July 2020 Mw 7.8 earthquake in the Shumagin Gap, to clarify shallow slip behaviour and the upper boundary in terms of fluid distribution. The authors successfully imaged the resistivity section in the near-trench forearc region to a depth of 16km, revealing trench-normal heterogeneity in the prism and insufficient fluid to explain the weak plate coupling.

The topic is interesting, and the objective is clear. The measurement methodology and inversion schemes are state-of-the-art, and the resistivity model is robust.

The Shumagin Gap is known to have a weakly coupled interface from geodesy, but the fluid distribution at the Gap has been debated in seismology and magnetotellurics. The present paper reports that the near-trench sedimentary fluid supply does not extend to greater depths within the plate interface. This conclusion is essential and casts new questions about the weakly coupled plate interface to the Shumagin Gap.

We thank the reviewer for the positive remarks. We appreciate the recognition of our work's novelty and contributions.

The reviewer has the following comments.

Regarding the influencing factor in plate coupling, fluid is a candidate. However, the existence of a seamount or the topography (plate-bending geometry) of the downgoing plate is another. Can the authors say whether the Shumagin Gap lacks seamounts or has a smoother plate geometry than the surroundings?

We thank the reviewer for this comment regarding alternative controlling mechanisms for plate coupling. The Shumagin Gap is indeed characterized by rough plate interface (Bécel et al., 2017; Li et al., 2018) and extensive bend faulting at the incoming plate (Shillington et al., 2015). Although specific seamounts are not clearly resolved, the overall lower-plate relief is rugged. We have revised the text (Line 46-51) to explicitly describe this topographic roughness. This roughness is a critical component of the "interplay" we conclude: it is the interplay of the whole system, rather than simply the fluids on the plate interface, that modulates the megathrust in this region.

Is there any other unique geophysical nature (excluding the geodesy) of the Shumagin Gap, as compared with the surrounding past-rapture area (M9 class)? To this reviewer, the Shumagin Gap is not as earthquake-prone as the surrounding areas, as it has had multiple M7-class earthquakes, as shown in Figure 1.

The Shumagin Gap was identified as a seismic gap since it has not ruptured in a great earthquake (Mw > 8) for at least a century (Davies et al., 1981). Excluding geodetic observations, several unique geophysical and geological features distinguish the Shumagin Gap from the surrounding regions or segments:

- Abundant interplate and intermediate-depth seismicity is observed at the Shumagin Gap, whereas the Semidi segment has relatively sparse seismicity (Shillington et al., 2015).
- Extensive plate bending-related faulting at incoming plate (Clarke et al., 2024; Shillington et al., 2015) and a rough lower-plate relief (Bécel et al., 2017; Li et al., 2018).
- The lack of a thick sediment supply (Li et al., 2018; Shillington et al., 2015).

While we briefly mentioned the Shumagin Gap's unique geophysical nature in the original manuscript, we have expanded this description in the revised version to clearly highlight other unique characteristics (Lines 46-49).

As trench-normal heterogeneity exists, the deeper part of the plate (where M7-class ruptures occurred) may contain fluids and have triggered the rupture. Will this idea conflict with the previous MT result?

This idea does not conflict with the previous collocated MT results (Cordell et al., 2023). In fact, the MT model explicitly images a conductive anomaly at depths > 25 km, which supports the reviewer's suggestion that fluids are present in the deeper part of the plate interface where M7-class ruptures have occurred. Crucially, the same MT model shows a resistive structure at depths shallower than 25 km. This independent constraint agrees well with our CSEM results, which infer a fluid-poor (low porosity) environment along the

shallow plate interface. These are illustrated in the newly added Fig. 4 and Fig. S3, which are discussed in detail in next response. Please also see our response to Reviewer #2.

The concluding figure, together with the previous magnetotelluric results, should emphasize the conclusion.

We appreciate this suggestion to visualize our findings in the context of previous studies. We have added Fig. 4 (Line 331-344, 276, 311, 355) as a new conceptual model, which combines the MT model (Cordell et al., 2023) with our CSEM results into a unified schematic. It clearly illustrates the heterogeneous forearc structure, emphasizing our conclusion.

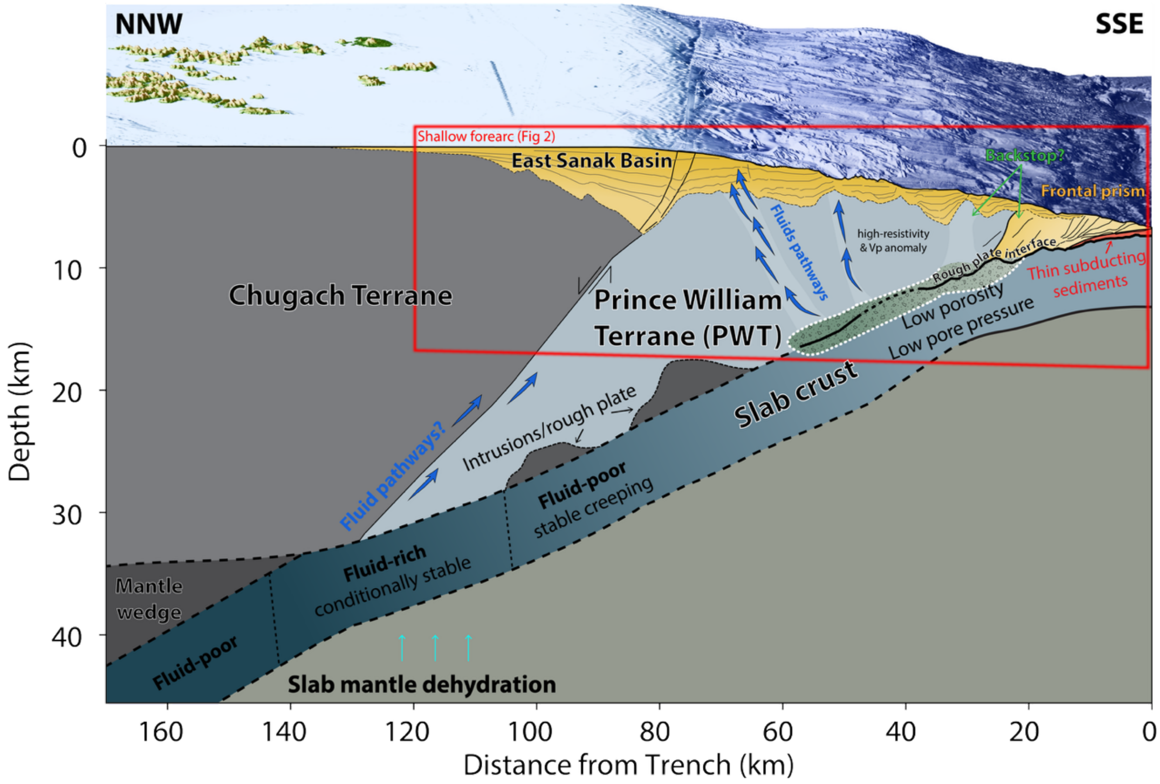


**Fig. 4. Schematic diagram illustrating a heterogeneous forearc and a fluid-poor shallow megathrust with low fluid pressure in the Shumagin Gap.** The red box outlines the schematic interpretation derived from the electrical resistivity model in Fig. 2a. The shallow megathrust is characterized by thin and irregular subducted sediments and a rugged plate interface. The interface is depicted as a diffuse fractured zone, incorporating the base of the overriding plate and the uppermost down-going oceanic crust, where dipping conductive fault zones (blue arrows) likely facilitate upward fluid migration. The observed fluid-starved distribution and inferred low fluid pressures indicate that elevated pore pressure cannot explain the low geodetic locking in this region. Instead, the structural complexity highlighted by the pronounced electrical resistivity heterogeneity both along the plate interface and within the overriding plate suggests

that the intricate interplay among fluids, geological structures, and dynamic processes controls the region's seismogenic behavior. Structural interpretations outside the red box are based on Cordell et al. (2023).

We have also included a supplementary figure (Fig. S3) to show the comparison between previous MT and our CSEM resistivity model together. This comparison highlights the consistency between the two independent datasets, thereby strengthening the robustness of our interpretation and conclusions. Please note that the sequence of supplementary figures and corresponding citations have been updated accordingly.

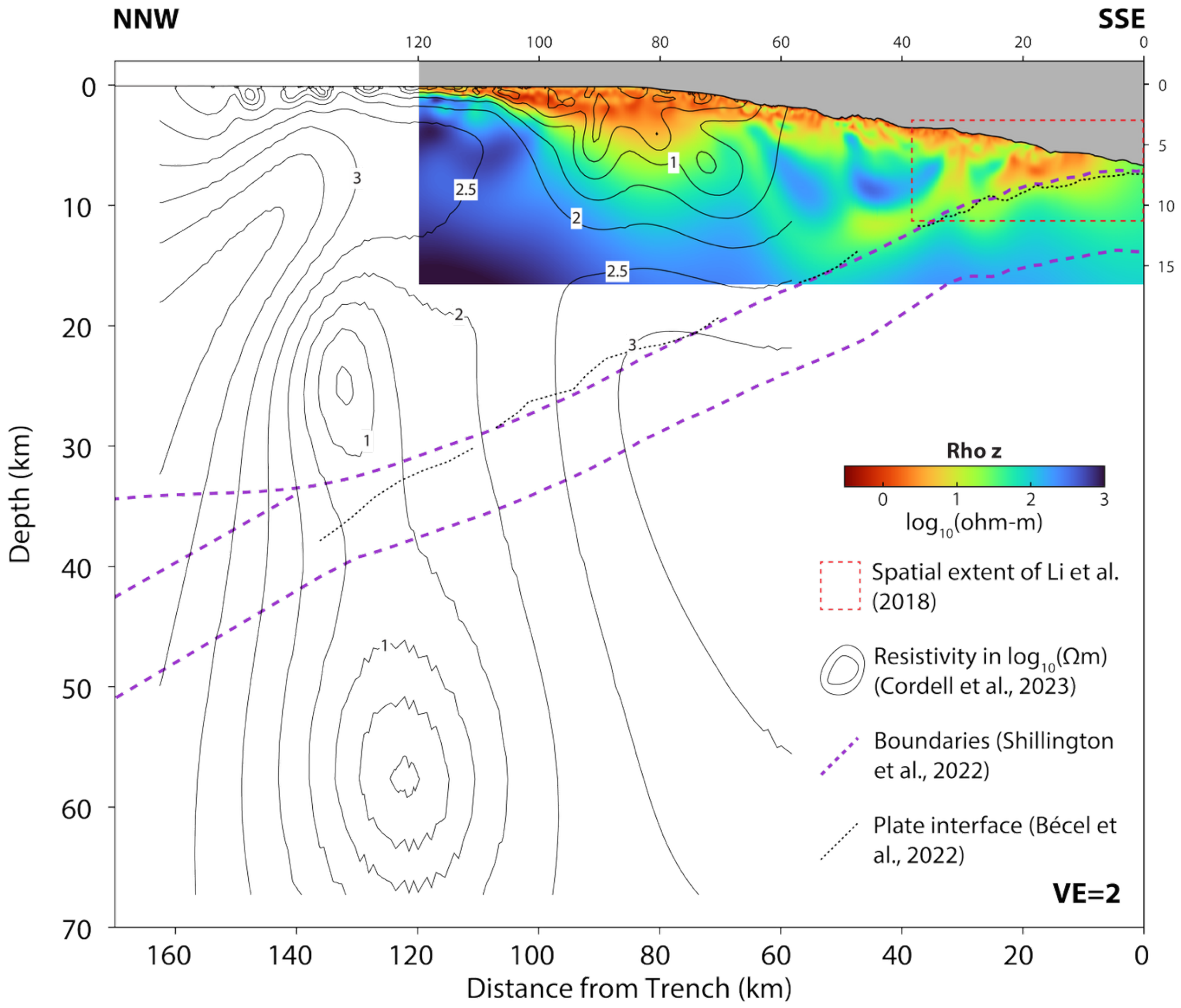


**Fig. S3. Comparison between our preferred resistivity model and the MT result (Cordell et al., 2023).** Background model is our preferred resistivity model shown in Fig. 2 and Fig. S1. Black solid lines are the contour line at 0.5 log unit intervals from the resistivity model from (Cordell et al., 2023). Red dashed rectangle outlines the spatial extent of P-wave velocity model from (Li et al., 2018). Black dashed line indicates the plate interface from (Bécel et al., 2017). Purple dashed lines denote slab crust boundaries from Shillington et al. (2022). The uppermost segment located at >135 km represents the overriding plate Moho.

In the future, another CSEM profile over the surrounding locked segments (e.g., Semidi) will directly clarify the authors' argument for water-starved subduction at the Shumagin Gap. Instead, the supply of fluids for the "locked" segment surrounding the Shumagin Gap may have triggered the large ruptures.

A graphical comparison or compilation of past similar studies (e.g., Nicaragua, Hikurangi) on the strongly/weakly coupled interface and the incoming sediments near the trench will strengthen the authors' argument.

This is a valid point. However, these comparisons present significant interpretative challenges. For the Nicaragua margin, while high porosities have been observed, the locking state of the shallow megathrust remains unconstrained due to a lack of seafloor geodetic data. Additionally, the region's history of anomalous tsunami earthquakes complicates the relationship between porosity and interplate coupling. Regarding the Hikurangi margin, the resistivity structure is highly heterogeneous, making it difficult to reliably identify the plate interface for comparable porosity estimation.

Nevertheless, to address the reviewer's point, we have compiled a comparison figure here (Fig. R1) plotting porosity estimates along the plate interface against depth below the seafloor. To minimize bias and maximize comparability between the datasets, we standardized the axis to depth below the seafloor rather than distance from the trench. As shown in Fig. R1, porosity estimates at the Shumagin Gap are significantly lower than those observed at the Nicaragua margin (Naif et al., 2016), which align closely with the global average trends (Bray and Karig, 1985). We excluded Hikurangi because in that study area the resistivity structure is highly heterogeneous, making it challenging to identify the plate interface where a porosity can be estimated. Therefore, given the uncertainties regarding the locking state in Nicaragua and heterogeneity in Hikurangi, we think sticking with the comparison to the global average values from Bray and Karig (1985) is sufficient to ensure a more robust and unbiased baseline for our study region (Fig. 3).

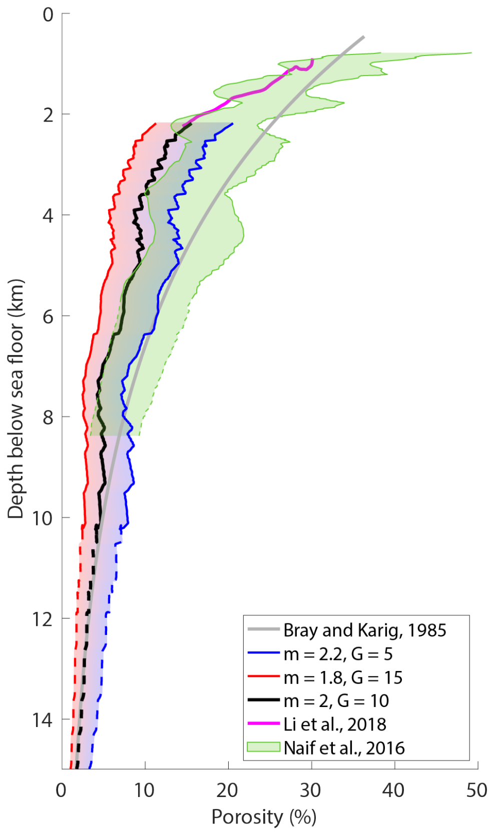


**Fig. R1. Comparison of porosity estimates along the plate interface versus depth below the seafloor.** The green shaded area represents porosity estimates derived from CSEM data at the Nicaragua margin (Naif et al., 2016). The other curves represent data from this study, following the same conventions as Fig. 3. Note that to facilitate direct comparison between margins, the vertical axis represents the depth below the seafloor, differing from the distance-to-trench axis used in the main text (Fig. 3).

We acknowledge the importance of placing our results in the context of other marine EM studies. Fortunately, the recent review by Chesley (2025), which has been cited in our revised manuscript (ref 54), offers a comprehensive summary of global marine EM findings. A compilation study specifically focused on forearc regions for these EM studies in the future would be the most appropriate venue to investigate the relationship between interseismic coupling state and fluids across different margins.

Regarding the interpretation of the porosity from the resistivity, how about the influence of clay minerals, like smectite? How is the fracture porosity?

The presence of conductive clay minerals (e.g., smectite) would contribute to the bulk conductivity via surface conductivity [see Naif et al. (2016) and Chesley et al. (2023) for further discussion on this effect]. If we were to account for this additional conductive mechanism, the fluid-filled porosity required to explain the observed resistivity would be

lower than the estimates derived from the Archie's law. Therefore, our current estimates represent an upper bound (or a conservative maximum) for porosity. Since our study focuses on the lack of fluid or low porosity along the plate interface, using this upper-bound estimate ensures our conclusions are conservative and robust.

Regarding the fracture porosity, if we acknowledge the presence of fractures, the cementation exponent should be lower. Similarly, the bulk porosity required to match the observed resistivity would again be lower than what we calculated using standard cementation exponent ($m \approx 2$).

We have clarified this in the revised manuscript (Line 535-540).

Specific comments:

Fig.1: The contour lines of the slab depth are shown with a 10km interval, but the depths should also be annotated in the figure.

Line 110-124 (Fig. 1): Thanks for helping improving readability of Fig. 1. We have explicitly annotated the depth values on the Slab2.0 contours (in km below sea level). To further visualize the slab interface, as also requested by Reviewer #3, we have incorporated the Slab2.0 interface into Figure 2a and 2c (Line 171).

Fig. 1 and L. 283: The SSEs can be mapped in Fig. 1.

The SSE patches identified in recent studies (He et al., 2023) are located in the Semidi segment, which lies to the northwest of our survey area. Since these events fall outside the spatial extent of Fig. 1, we are unable to display them on the current map.

Fig.3 Caption: Lambda should be defined, or the definition should be referenced.

We have referenced the definition of $\lambda^*$ (Line 543 in Methods) in Fig. 3 caption (Line 212).

## Reviewer #2

This manuscript presents a new 2-D high-resolution image of electrical resistivity in the Shumagin seismic gap, using the latest collected controlled-source electromagnetic data from the seafloor. The authors further interpret the low-resistivity anomalies as rock porosity filled with fluids. The results show that the plate interface in the top 10 km has a lower porosity than previously expected. This is inconsistent with previous suggestions that abundant fluids on the plate interface result in a weak interseismic plate coupling in the Shumagin Gap. The authors suggest that it is the interplay of the whole system, rather than simply the fluids on the plate interface, that modulates the megathrust in this region.

Since electromagnetic data analysis is not my expertise, my comments mainly focus on interpretations and defer the technical part to other reviewers.

I think this is an exciting discovery. It, together with some recent studies, challenges a long-standing simplified model that the fluids on the plate interface dominate the megathrust. I recommend a minor revision. Here are my comments, as the authors had addressed many of my early concerns.

We are grateful for the reviewer's positive remarks and recognition of our work's novelty and contributions. We believe the comments have further improved the manuscript.

Line 39: The hypothesis needs a reference, ideally in rock physics.

Line 41: We have added two references (Cappa et al., 2019; Scuderi et al., 2017) to support the hypothesis.

Line 59: I suggest adding "..., although the shallow portion (<10 km) of the megathrust near the trench remains poorly imaged."

Line 64-65: Agreed. We have accepted this suggestion to improve the precision of our description of Wang et al. (2024).

Lines 260–276: These sentences need to be more precise on the depths. Earthquake tomography (Wang et al., 2024) and MT images (Cordell et al., 2023) show fluids on the interface deeper than 25 km, but CSEM (this study) and active-source seismic imaging (Li et al., 2018) show a fluid-starving interface shallower than 25 km. In contrast, interseismic plate coupling models usually extend to 45 km depth, and have very low resolution near the trench (<15 km depth). So do the authors propose that the entire megathrust weak coupling is not related to fluids on the interface or only the shallow portion of the megathrust? Please be more specific on the depths.

We have rewritten portions of this paragraph (Line 270-287) to be precise about depth. We agree that our CSEM data primarily constrains the shallow interface. We would like to clarify that while the MT model (Cordell et al., 2023) shows a conductive segment at depths > 25 km (attributed to slab mantle fluids), the model becomes resistive again farther downdip, rather than indicating continuous fluids all the way along the deep interface (see Fig. S3 and newly added Fig. 4, in responses to Reviewer #1). The MT results also show a resistive region updip of 25 km indicating a lack of fluids, consistent with the shallower CSEM result (Fig. S3). By integrating the MT, CSEM, and active-source seismic imaging results, we can infer the fluid distribution along the plate interface from the trench axis down to 40 km depth. Based on the cumulative result, we suggest that fluids are

unlikely to be the sole or primary governing factor for the megathrust locking state across the entire megathrust in the Shumagin Gap.

## Reviewer #3 Kelin Wang

Although I am unable to scrutinize fine technical details of the data collection and processing reported in this paper, there is enough information in the paper to help me assess the validity and importance of the scientific story. As far as I can tell, the data and the research work are of high quality, and the scientific conclusions are important. The paper thus deserves timely publication in Nature Communications with some minor revision. I am returning an annotated pdf copy of the manuscript marked with my comments. I am only reiterating a few of them here.

We are grateful for the reviewer's encouraging assessment of our work and the recognition of the high quality of the marine controlled-source EM data and the importance of this study. We have carefully reviewed the annotated PDF and incorporated the suggestions into the revised manuscript. We believe that these constructive comments have helped improve the clarity and precision of the manuscript.

The title of the paper reads like that of a newspaper report. This is unfortunately what journals like Nature and Science are promoting today. I wish these journals could understand that objectivity is the lifeline of science, and an assertion or claim is inappropriate for the title of a scientific paper. I don't know what the authors can do about it given the (strangely) tight word limit imposed by these journals, but to the minimum I think readers deserve to know from the title what scientific approach is used (e.g., electromagnetic imaging, or at least geophysical imaging).

We appreciate the reviewer's perspective on scientific objectivity of the title. Given the maximum title length (15 words), we have changed the title to "Electromagnetic imaging reveals insufficient fluids to explain shallow megathrust creep at the Shumagin Gap"

The meaning of "weak coupling" is unclear. Obviously, this paper is trying to address whether fluid controls slip behavior, creeping vs. stick-slip. A decrease in effective normal stress leads to a lower shear strength, but it does not translate to slip behavior. See Wang and Dixon (2004, https://doi.org/10.1029/2004EO180005) and Wang and Bilek (2014, ref 45 in this manuscript)). Instead of weak or strong coupling, I suggest that clear wording is used as needed such as "lower shear stress" (force), "velocity-strengthening" (property or behavior), "creeping" (kinematics), etc.

We appreciate this terminology suggestion. By weak coupling, we meant specifically the geodetic locking constrained in this region, which show values near zero indicating that the

subducting slab is creeping. Acknowledging the potential ambiguity between mechanical strength and kinematic behavior, we have adopted the reviewer's suggestion and have replaced "weak (plate) coupling" with "(megathrust) creep" or "low geodetic locking" throughout the manuscript (Title, Line 29, 41, 67, 83, 253, 274, 362). We have also replaced "plate coupling" with "megathrust locking (state)" (Line 24, 57, 71, 250, 288, 308, 358, 367).

I suggest that the authors make a better effort in linking figures to text and connecting different figures. See comments in the annotated manuscript.

We have gone through the manuscript and improved the cross-referencing between the main text and the figures. Here is a quick summary. Details can be seen in point-by-point response regarding each figure.

1. As also recommended by Reviewer # 1, we have added labels of Slab2.0 model (Hayes et al., 2018) in Fig. 1 and its geometry in Fig. 2.
2. We have marked the profile (thick orange line) in Fig. 1 to show the site range in Fig. 2a to link map and model better.
3. We have revised Figs 1 and 2's captions to consistently use the term "OBEM receiver" for stations to keep a better link between Fig. 1 and Fig. 2.

To me, having pore fluid pressure lower than hydrostatic is not physically unrealistic, although one can consider it "unlikely". I made some suggestion to rephrase line 222.

Line 221-222: Sub-hydrostatic pore fluid pressure is not physically unrealistic. Perhaps "unlikely" is a better word. Then the rest of the sentence should be less definitive: "which may be an artifact ...".

Line 230-231: We have accepted the suggestions and rewritten this sentence in the revised manuscript.

In the discussion of applicability of the sediment compaction model in lines 221-226, only the presence basalt is mentioned. I made a suggestion to consider sediment deformation (shear vs. in situ compaction).

Line 225-226: Composition is just one aspect. Deformation is also an issue. Subducted sediment likely endures much shear deformation, very different from sediment that only endures compaction.

We agree that there are many possible complicating factors besides composition. We now point this out, but we prefer not to get into expand the discussion or get into specifics since these factors are challenging to constrain with our observations and therefore beyond the scope of our study. We have revised the text to explicitly acknowledge shear deformation

as an unconstrained complicating factor when discussing the applicability of the sediment compaction model (Line 234-236).

**Other Annotated PDF comments**

We are thankful for the reviewer's detailed editorial suggestions marked in the annotated PDF. We have accepted these suggestions to improve the clarity and flow of the text.

Line 51: ? Sounds like all intraslab seismicity is seaward of the arc.

Line 55: We have deleted "subarc".

Fig.1

- Please mark the range of the section in Fig. 2a.

To clarify the location of the section shown in Fig. 2, we have added a profile line on the map figure (Fig. 1). Correspondingly, we have added a sentence "The thick orange line marks the location and extent of the cross-section shown in Fig. 2a." in Fig. 1's caption.

- Please label a few of them on map. Also, please explain whether the depth is below sea level or seafloor.

We have added labels of Slab2.0 depth contours in Fig. 1. The depth is below sea level.

Line 140: Not an accurate description given the big blue patches seaward of it. Also, "rigid block" means infinite rigidity which neither is true nor can be inferred from resistivity signals.

Line 149-151: We have rephrased and clarified this sentence.

Line 144: Do we know the rock types to make this statement? Or is it an inference based on some reference? Also, if this thing marks this separation, what do the backstops marked in Fig. 2a do?

Line 151-152: We clarify that this statement is an inference based on the distinct contrast in high velocity/resistivity vs. low velocity/resistivity. The high-resistivity anomaly (R1) is interpreted as the older and stronger framework materials. So, the boundary between R1 and the conductive frontal prism spatially corresponds to the backstops marked in Fig. 2a. To resolve the confusion and align the text with the figure, we have revised the sentence to "The sharp contrast between R1 and conductive frontal prism suggests a boundary separating the strong, older framework materials from the younger frontal prism."

Fig.2

- Line 149: Please label this feature in Fig. 2 and quote the figure here.

Line 157: We have labeled this feature in Fig. 2 and quoted the figure here.

- Please also show Slab2.0 (shown in Fig. 1) to allow the reader to build a connection with Fig. 1.

Line 171: Agreed. We have added the Slab2.0 interface and labeled it in the revised Fig. 2. We have updated the caption accordingly.

- Please make a better link with Fig. 1 by using the same circle color and same description for stations (e.g., Fig. 1 does not mention OBEM).

We didn't update the color of receivers in Fig. 2. But we have revised Figs 1 and 2's captions to consistently use the term "OBEM receiver" instead of "station" that was used in Fig. 1's caption previously. We have corrected this to avoid confusion as they represent the same sites.

Line 185: Please use a separate sentence to clarify meaning.

Line 193: We have removed this part to increase clarity.

Line 193: in the Semidi segment? If so, I suggest to use clearer writing like "Even if we extrapolate ... at the Semidi segment, the inferred ... would still be higher than the near-trench ... for our study area."

Line 200-201: We thank the reviewer for pointing out this potential ambiguity. We would like to clarify that the "generic porosity compaction trend" refers to a global averaged compaction model (Bray and Karig, 1985), rather than the specific trend from the Semidi segment. To prevent this confusion for future readers, we have added "global" and used the connector "Furthermore" in revised sentence: "Furthermore, extrapolating a global generic porosity compaction trend (Bray and Karig, 1985) towards the seafloor as a lower bound still results in higher porosity values than the near-trench estimates obtained for this region."

Line 226-227: refer to Methods? Otherwise unexplained.

Line 237: Yes. We have added "(Methods)" to the sentence.

Line 261-262: This seems to imply that the fluids facilitated the 2020 earthquake. Is this what you mean? If not, revise the text to clarify.

Line 271: That was not our intension. We agree that the phrasing was misleading. To avoid any further confusion or misinterpretation, we have decided to delete this sentence in the revised manuscript.

Line 328: Please clarify whether you mean "we think ..." or the imaging can resolve this.

Line 353: We have rewritten the sentence in the revised manuscript.

## Other Changes

### Technical Correction

Supplementary Materials: We noticed a minor typo regarding the figure label in the Supplementary Materials where Fig. S1 was inadvertently labeled as "Fig. S6". The figure content, order, and its citation in the main text were correct. We have corrected this label in the revised Supplementary Information file.

Fig. 1: We corrected unit typo in colorbar of Fig. 1.

### Addition of Recent Event on Map

To ensure that the Fig. 1 provides the most up-to-date representation of the regional seismic activity, we have updated Fig. 1 to include the GCMT focal mechanism of the M7.3 strike slip Sand Point earthquake that occurred in July 2025.

### Colormap of Fig. S2

We have updated the colormap in Supplementary Fig. S2 (showing porosity) to distinguish it from the resistivity models in Fig. 2 of the main text to ensures that the two different physical properties (porosity vs. resistivity) are visually distinct to the reader.

## References

Bécel, A., Shillington, D.J., Delescluse, M., Nedimović, M.R., Abers, G.A., Saffer, D.M., Webb, S.C., Keranen, K.M., Roche, P.-H., Li, J., Kuehn, H., 2017. Tsunamigenic structures in a creeping section of the Alaska subduction zone. Nat. Geosci. 10, 609–613. https://doi.org/10.1038/ngeo2990

Bray, C.J., Karig, D.E., 1985. Porosity of sediments in accretionary prisms and some implications for dewatering processes. J. Geophys. Res. Solid Earth 90, 768–778. https://doi.org/10.1029/JB090iB01p00768

Cappa, F., Scuderi, M.M., Collettini, C., Guglielmi, Y., Avouac, J.-P., 2019. Stabilization of fault slip by fluid injection in the laboratory and in situ. Sci. Adv. 5, eaau4065. https://doi.org/10.1126/sciadv.aau4065

Chesley, C., 2025. Insights on Marine Tectonophysics from Recent Marine Electromagnetic Studies. Surv. Geophys. https://doi.org/10.1007/s10712-025-09903-w

Chesley, C., Naif, S., Key, K., 2023. Characterizing the porosity structure and gas hydrate distribution at the southern Hikurangi Margin, New Zealand from offshore

electromagnetic data. Geophys. J. Int. 234, 2411–2428. https://doi.org/10.1093/gji/ggad243

Clarke, J., Shillington, D.J., Regalla, C., Gaherty, J.B., Estep, J., Wiens, D.A., Bécel, A., Nedimović, M.R., 2024. Controls on Bending-Related Faulting Offshore of the Alaska Peninsula. Geochem. Geophys. Geosyst. 25, e2023GC011271. https://doi.org/10.1029/2023GC011271

Cordell, D., Naif, S., Evans, R., Key, K., Constable, S., Shillington, D., Bécel, A., 2023. Forearc seismogenesis in a weakly coupled subduction zone influenced by slab mantle fluids. Nat. Geosci. 16, 822–827. https://doi.org/10.1038/s41561-023-01260-w

Davies, J., Sykes, L., House, L., Jacob, K., 1981. Shumagin Seismic Gap, Alaska Peninsula: History of great earthquakes, tectonic setting, and evidence for high seismic potential. J. Geophys. Res. Solid Earth 86, 3821–3855. https://doi.org/10.1029/JB086iB05p03821

Hayes, G.P., Moore, G.L., Portner, D.E., Hearne, M., Flamme, H., Furtney, M., Smoczyk, G.M., 2018. Slab2, a comprehensive subduction zone geometry model. Science 362, 58–61. https://doi.org/10.1126/science.aat4723

He, B., Wei, X., Wei, M., Shen, Y., Alvarez, M., Schwartz, S.Y., 2023. A shallow slow slip event in 2018 in the Semidi segment of the Alaska subduction zone detected by machine learning. Earth and Planetary Science Letters 612, 118154. https://doi.org/10.1016/j.epsl.2023.118154

Li, J., Shillington, D.J., Saffer, D.M., Bécel, A., Nedimović, M.R., Kuehn, H., Webb, S.C., Keranen, K.M., Abers, G.A., 2018. Connections between subducted sediment, pore-fluid pressure, and earthquake behavior along the Alaska megathrust. Geology 46, 299–302. https://doi.org/10.1130/G39557.1

Naif, S., Key, K., Constable, S., Evans, R.L., 2016. Porosity and fluid budget of a water-rich megathrust revealed with electromagnetic data at the Middle America Trench. Geochem. Geophys. Geosyst. 17, 4495–4516. https://doi.org/10.1002/2016GC006556

Scuderi, M.M., Collettini, C., Marone, C., 2017. Frictional stability and earthquake triggering during fluid pressure stimulation of an experimental fault. Earth Planet. Sci. Lett. 477, 84–96. https://doi.org/10.1016/j.epsl.2017.08.009

Shillington, D.J., Bécel, A., Nedimović, M.R., 2022. Upper Plate Structure and Megathrust Properties in the Shumagin Gap Near the July 2020 M7.8 Simeonof Event. Geophys. Res. Lett. 49, e2021GL096974. https://doi.org/10.1029/2021GL096974

Shillington, D.J., Bécel, A., Nedimović, M.R., Kuehn, H., Webb, S.C., Abers, G.A., Keranen, K.M., Li, J., Delescluse, M., Mattei-Salicrup, G.A., 2015. Link between plate fabric, hydration and subduction zone seismicity in Alaska. Nat. Geosci. 8, 961–964. https://doi.org/10.1038/ngeo2586
